# Comparing efficacy and safety of low-dose versus standard-dose antiplatelet therapy in stroke patients: a meta-analysis

**DOI:** 10.3389/fphar.2024.1484130

**Published:** 2025-01-06

**Authors:** Zhao Ren, Chunxing Li, Xin Zhang, Lichaoyue Sun, Hui Zhu, Dongxiao Wang, Yumin Wang, Shuo Liang, Guanchun Wang

**Affiliations:** ^1^ Department of Pharmacy, Aerospace Center Hospital, Peking University Aerospace School of Clinical Medicine, Beijing, China; ^2^ Department of Respiratory and Critical Care Medicine, Aerospace Center Hospital, Peking University Aerospace School of Clinical Medicine, Beijing, China; ^3^ Department of Neurology, Aerospace Central Hospital, Peking University Aerospace School of Clinical Medicine, Beijing, China

**Keywords:** low-dose, antiplatelet therapy, stroke, myocardial infarction, bleeding, meta-analysis

## Abstract

**Background:**

Stroke is the leading cause of disability globally, with antiplatelet therapy being crucial for secondary prevention but also increasing bleeding risks. This requires careful dosage adjustments to balance thrombosis and bleeding risks.

**Objective:**

This study compared the efficacy and safety of low-dose versus standard-dose antiplatelet therapy in stroke patients.

**Methods:**

We conducted a comprehensive search across multiple databases, including PubMed, Embase, the Cochrane Library, ClinicalTrials.gov, CNKI, and the Wanfang Medical Database, up to March 2024. Only randomized controlled trials assessing low-dose antiplatelet therapy in stroke patients were considered. The Cochrane Risk of Bias Tool (RoB 2) was used for quality. Performed meta-analysis using Stata 15.0, with relative risk (RR) and 95% confidence interval (CI) as effect estimates.

**Results:**

Ten studies involving 7,703 Asia participants, mainly from China and Japan, were analyzed. The meta-analysis revealed that low-dose reduces the risk of bleeding (RR 0.51; 95% CI 0.27, 0.98) compared to standard dose, with similar risks for stroke (RR 1.04; 95% CI 0.69, 1.55), myocardial infarction (MI) (RR 1.91; 95% CI 0.88, 4.12), all-cause death (ACD) (RR 1.17; 95% CI 0.38, 3.62), and major bleeding (RR 0.74; 95% CI 0.16, 3.30). Subgroup analysis revealed that compared to standard-dose clopidogrel, low-dose clopidogrel increased the risk of MI. Notably, this increased risk was observed specifically within the Chinese population but not in the Japanese population. Low-dose clopidogrel and low-dose prasugrel reduce the risk of bleeding compared to standard-dose clopidogrel, but there is no statistically significant difference. Low-dose aspirin significantly reduces the risk of bleeding compared to standard-dose aspirin.

**Conclusion:**

In patients with stroke in Asia, low-dose antiplatelet therapy significantly reduces the risk of bleeding compared to standard doses, with consistent risks of stroke, MI, ACD, major bleeding, and discontinuation due to bleeding.

## 1 Introduction

Stroke affects people of all ages and has become the second leading cause of disability and mortality, imposing a significant burden on both individuals and society (Saini et al., 2021). Ischemic stroke constitutes over 80% of all stroke subtypes, representing most incidences (Kuo et al., 2017). It is also the leading cause of death in China and the fifth leading cause of death in the US (Del Brutto et al., 2019; Easton et al., 2009). According to the World Health Organization, 15 million people suffer from strokes worldwide each year (Minhas et al., 2022). In the US, the prevalence of stroke is approximately 3% in adults aged 20 years or older, accounting for approximately 7 million strokes in the population (Virani et al., 2020).

In the secondary prevention of stroke, in addition to controlling risk factors such as hypertension, diabetes, and dyslipidemia, the use of antiplatelet therapy has long been a consensus (Naqvi et al., 2020). Antiplatelet agents inhibit platelet activation and aggregation, reducing the risk of thrombotic events (Virk et al., 2023). For patients with non-cardioembolic ischemic stroke and transient ischemic attack (TIA), antiplatelet therapy can significantly reduce the risk of major adverse cardiovascular events (MACE), including non-fatal stroke, non-fatal myocardial infarction, and vascular-related death (Antithrombotic Trialists’ Collaboration, 2002). Antiplatelets modify the risk of future stroke events and reduce the deathrate in the acute and the-long term periods (Sandercock et al., 2014). In individuals with ischemic stroke or TIA, long-term antiplatelet therapy prevents about 36 serious vascular events for every 1,000 patients treated for 3 years (Antiplatelet Trialists' Collaboration, 1994, Antithrombotic Trialists’ Collaboration, 2002). Thus, lifelong antiplatelet drug treatment is recommended after TIA or non-cardioembolic ischemic stroke, according to international and national guidelines (Kernan et al., 2014).

An effective secondary prevention strategy is crucial to reduce recurrence, disability, and mortality in patients (Powers et al., 2018). Antiplatelet drugs are widely applied in the secondary prevention of cardiovascular disease. Although they successfully reduce the risk of recurrent ischemic events (Baigent et al., 2009), antiplatelet drugs are associated with a small but significant risk of serious bleeding, reported to vary between 1% per year and 1.5% per year (Alberts et al., 2011; Li et al., 2017). Among patients enrolled in long-term controlled trials of antiplatelet agents for cardiovascular prophylaxis, low-dose aspirin increases the risk of major bleeding two times compared to placebo (McQuaid and Laine, 2006). The rate of major bleeding was higher in the aspirin group than in the placebo group (3.8% vs. 2.8%; hazard ratio, 1.38; 95% confidence interval, 1.18 to 1.62; *P* < 0.001) (McNeil et al., 2018). Aspirin use was associated with an increased risk of major bleeding events compared to without aspirin (23.1 per 10,000 participant-years with aspirin and 16.4 per 10,000 participant-years without aspirin) (Zheng and Roddick, 2019).

Bleeding complications may offset the benefit of antiplatelet drugs (Hilkens et al., 2021). Therefore, optimizing drug dosage to strike a delicate balance between thrombosis risk and bleeding is crucial to maximize treatment efficacy for patients with stroke. The exploration of efficacy and safety of low-dose antiplatelet drugs is limited, with varying definitions of dose ranges and frequent inclusion of patients with various cardiovascular conditions along with strokes (Gouya et al., 2014; McQuaid and Laine, 2006). Some studies also used placebos as controls (McQuaid and Laine, 2006; Niu et al., 2016). This meta-analysis aimed to evaluate the efficacy and safety of low-dose antiplatelet drugs compared to standard-dose antiplatelet drugs in stroke patients.

## 2 Methods

This meta-analysis followed the Preferred Reporting Items for Systematic Reviews and Meta-Analyses (PRISMA) guidelines (Page et al., 2021). The study protocol was registered with the International Prospective Register of Systematic Reviews (PROSPERO) under registration number CRD42024556992.

### 2.1 Data sources

A comprehensive search was conducted for relevant literature across PubMed, Embase, the Cochrane Central Register of Controlled Trials, China National Knowledge Infrastructure (CNKI), and the Wanfang Data Knowledge Service Platform. The search strategy was developed with a medical information specialist (Supplementary S1). Two independent researchers, blinded to each other’s assessments, conducted the screening process using EndNote X9. Discrepancies were resolved by a third researcher. Reference lists of relevant systematic reviews were assessed to identify additional studies. We also searched trial registries, such as ClinicalTrials.gov, for ongoing or unpublished trials. The search covered database inception to 27 March 2024. Studies published in English and Chinese were considered for inclusion.

### 2.2 Study selection

#### 2.2.1 Participants

The studies included participants aged 18 years or older, clinically diagnosed with stroke, with no restrictions on sex or race.

#### 2.2.2 Interventions and controls

The intervention group received low-dose antiplatelet drugs (aspirin, indobufen, clopidogrel, prasugrel, ticagrelor, cilostazol, and dipyridamole), while the control group received standard-dose or low-dose antiplatelet drugs. The “low dose” categorization was based on predefined cut-off points derived from product characteristics summaries and clinical trial dosing regimens. The detailed dose classifications are summarized as follows:

#### 2.2.3 Standard-dose antiplatelet drugs

Aspirin: 100 mg/d; Indobufen: 200–400 mg/d, for patients aged 65 and older, 100 mg/d; Clopidogrel: 75 mg/d; Prasugrel: for patients weighing ≥60 kg and aged <75 years, 10 mg/d, for patients weighing <60 kg or aged ≥75 years, 5 mg/d; Ticagrelor: maintenance dose is twice daily, 180 mg/d, for patients with a history of myocardial infarction for at least 1 year and at least one high-risk factor for atherosclerotic thrombotic events or high-risk atherosclerotic thrombotic events in acute coronary syndrome (ACS), 60 mg/d; Cilostazol: 0.2 g/d; Dipyridamole: 75–150 mg/d.

#### 2.2.4 Low-dose antiplatelet drugs

Aspirin: 50 or 25 mg/d; Indobufen: 100 mg/d; Clopidogrel: 50 or 25 mg/d; Prasugrel: for patients weighing ≥60 kg and aged <75 years, 5 or 3.75 mg/d, for patients weighing <60 kg or aged ≥75 years, 3.75 mg/d; Ticagrelor: 120 or 90 mg/d. For patients with a history of myocardial infarction for at least 1 year and at least one high-risk factor for atherosclerotic thrombotic events or high-risk atherosclerotic thrombotic events in ACS, 90 or 60 mg/d; Cilostazol: 50 or 100 mg/d; Dipyridamole: 50 mg/d.

#### 2.2.5 Outcomes

The primary efficacy outcomes were the risk of ischemic stroke and MI. The primary safety outcomes were the occurrence of bleeding events and major bleeding. Additional outcomes included MACE, cardiovascular death (CVD), all-cause death (ACD), bleeding events leading to discontinuation, and life-threatening bleeding. MACE was defined as a composite of CVD, nonfatal MI, and nonfatal stroke. Major bleeding and life-threatening bleeding were defined according to the Thrombolysis in Myocardial Infarction (TIMI) criteria or the Bleeding Academic Research Consortium (BARC) criteria (Mehran et al., 2011). Major bleeding was defined by TIMI criteria or type 3b according to BARC criteria.

#### 2.2.6 Studies

The study included randomized controlled trials (RCTs). Efficacy outcomes required a minimum follow-up of 6 months, while safety outcomes required at least 1 month of follow-up.

### 2.3 Data extraction

A preliminary pilot study collected information using a validated standardized form. This form captured study details, including participating countries or regions, participant status, follow-up duration, patient numbers, and reported events. Patient demographic data, such as average or median age, percentage of female participants, and baseline body mass index or weight, were also recorded. Data related to interventions focused explicitly on low-dose platelet aggregation inhibitors. Two reviewers conducted all data extraction procedures, which were reviewed and confirmed by another reviewer.

### 2.4 Quality assessment

Two investigators independently assessed the quality of the studies using the revised Cochrane Risk of Bias Tool for Randomized Trials (RoB 2.0) (Sterne et al., 2019). This method considers the study’s randomization process, bias due to deviations from the intended interventions, missing outcome data, outcome measurement, and selection of the result reported. Each domain was qualitatively categorized as having a high, moderate, or low risk of bias.

### 2.5 Statistical analysis

The meta-analysis was conducted using Stata version 15.0 software (StataCorp, College Station, TX, United States). Statistical heterogeneity between studies was evaluated using the *I* ^*2*^ statistic. A random-effects model was applied if *I* ^*2*^ > 50% or P < 0.05; otherwise, a fixed-effects model was used. Categorical variables were expressed using the relative risk (RR) and 95% confidence intervals (CI), with a P-value less than 0.05 considered statistically significant.

Sensitivity analysis systematically excluded one study at a time to ensure the stability of the results. Publication bias was assessed qualitatively using contour-enhanced funnel plots and quantitatively using Egger’s test. Contour-enhanced funnel plots highlight areas of statistical significance and differentiate publication bias from other sources of asymmetry. A P-value less than 0.05 in the Egger test indicates potential publication bias.

## 3 Results

### 3.1 Literature retrieval and selection

**FIGURE 1:**
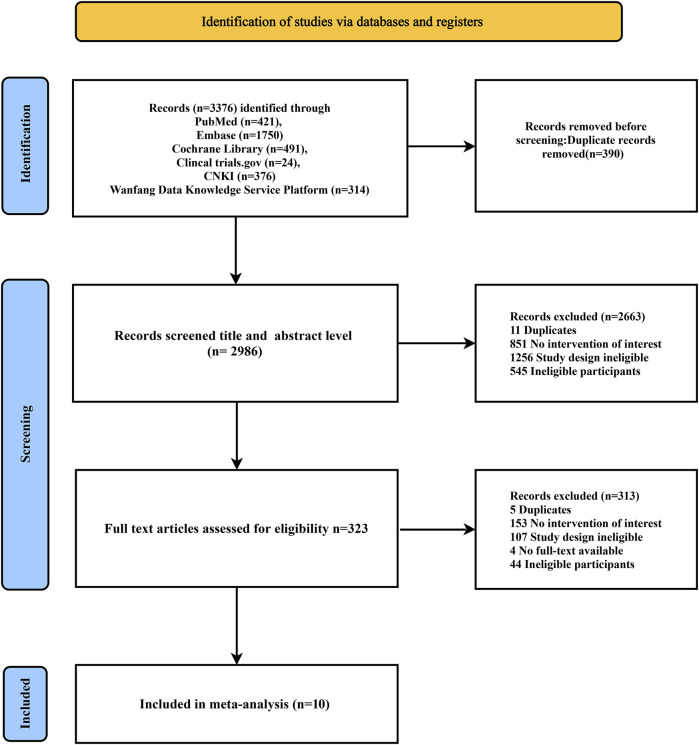
The literature search and selection process.

The search process identified 3,376 articles. After screening the titles and abstracts of 2,986 articles, 2,663 were found to be irrelevant and excluded. This left 323 articles for a detailed full-text review. Of these, 10 RCTs (n = 7,703) (Kitagawa et al., 2020; Kitazono et al., 2023; Ogawa et al., 2019; Uchiyama et al., 2012; Zuo et al., 2017; Liu Fan et al., 2014; Qingbo, 2015; Xinhua, 2016; Youtao, 2005; Yuehua, 2006) (Supplementary S2) met the inclusion criteria. Figure 1 shows the literature screening process.

### 3.2 Study characteristics and quality assessment

Five RCTs were in English (Kitagawa et al., 2020; Kitazono et al., 2023; Ogawa et al., 2019; Uchiyama et al., 2012; Zuo et al., 2017), and five were in Chinese (Fan et al., 2014; Qingbo, 2015; Xinhua, 2016; Youtao, 2005; Yuehua, 2006). The low-dose group included 4,019 patients, while the standard-dose group comprised 3,684. The studies spanned from 2005 to 2023, with two published in 2020 or later. Six studies (60.00%) included Chinese participants, four studies (40.00%) included Japanese participants. The proportion of women ranged from 19.6% to 48.3%. The median duration of the intervention period was 52 weeks (12–260 weeks). Details are shown in Table 1.

**TABLE 1:** Characteristics of included studies.

Trial registration	Country	Population	Duration	Outcome	Randomised treatments + dose	Drug class	No of Patients Randomized	Age	Female (%)	Mean BMI (kg/m^2^)
Kitazono et al. (2023)	Japan	thrombotic stroke	24–48 weeks	MACE/MI/Ischaemic stroke	Prasugre l3.75 mg QD	low	120	70.5 ± 9.38	28.8	62.81 ± 10.96
				/Bleeding/Major bleeding/						
				bleeding events leading to discontinuation						
				/life-threatening bleeding	Clopidogrel 75 mg QD	standard	114	70.0 ± 9.50	29.5	64.35 ± 11.36
Kitagawa et al. (2020)	Japan	non-cardioembolic ischemic stroke	1 year	MACE/MI/Ischaemic stroke	Prasugrel 3.75 mg QD	low	216	76.1 ± 7.6	44.9	55.0 ± 8.9
				/Bleeding/Major bleeding/	Prasugrel 2.5 mg QD	low	215	76.7 ± 7.0	43.7	55.9 ± 9.1
				bleeding events leading to discontinuation/life-threatening bleeding	Clopidogrel 50 mg QD	low	223	76.4 ± 7.3	43	56.0 ± 9.7
Ogawa et al. (2019)	Japan	non-cardioembolic stroke	96–104 weeks	MI//Ischaemic stroke/Major bleeding/bleeding events leading to discontinuation	Prasugrel 3.75 mg QD	low	1885	61.9 ± 8.7	20	65.8 ± 10.5
				/life-threatening bleeding	Clopidogrel 75 mg QD	standard	1862	62.4 ± 8.4	22	65.4 ± 9.7
Zuo et al. (2017)	China	ICVD combined with intracranial and extracranial arteriostenosis	12 weeks	Ischaemic stroke/CVD/ACD	Clopidogrel 50 mg QD + Aspirin100 mg QD	low	66	61.58	42.4	-
					Clopidogrel 75 mg QD + Aspirin 100 mg QD	standard	66	61.55	36.4	-
Uchiyama et al. (2012)	Japan	noncardioembolic ischemic stroke/ischemic stroke	52 weeks	MI/Ischaemic stroke	Clopidogrel 50 mg QD	low	558	62.28 ± 8.0	20.4	64.28 ± 8.9
					Clopidogrel 75 mg QD	standard	552	62.08 ± 8.6	19.6	65.38 ± 9.6
Youtao (2005)	China	ICVD	5 years	Ischaemic stroke/ACD/Bleeding	Aspirin 50 mg QD	low	302	58 ± 9.2	45.4	-
					Aspirin 300 mg QD	standard	211	60 ± 8.8	48.3	-
Yuehua (2006)	China	IMS/RIND/MS	1 year	Ischaemic stroke	Aspirin 50 mg QD	low	30	NA	NA	-
					Aspirin 100 mg QD	standard	30	NA	NA	-
Fan et al. (2014)	China	ischemic stroke	52 weeks	MI/Ischaemic stroke/Bleeding	Clopidogrel 50 mg QD	low	558	62.2 ± 8	20.4	64.2 ± 8.6
					Clopidogrel 75 mg QD	standard	552	62 ± 8.6	19.6	65.3 ± 9.6
Qingbo, B (2015)	China	acute cerebral infarction	1.5 years	MI/Ischaemic stroke/ACD/Bleeding	Clopidogrel 50 mg QD	low	39	59.93 ± 10.78	35.9	-
					Aspirin 100 mg QD	standard	45	58.44 ± 10.19	31.1	-
Xinhua (2016)	China	acute cerebral infarction	24 weeks	Bleeding	Aspirin 50 mg QD	low	30	59.8 ± 7.09	46.7	-
					Aspirin 100 mg QD	standard	29	60.0 ± 6.91	41.4	-

ICVD, ischemic cerebrovascular disease; TIA, transient ischemic attack; IMS, intermittent monocular scotoma; RIND, reversible ischemic neurologic deficit; MS, minor stroke; BMI, body mass index; QD, quaque die; BID, bis in die; -, not applicable.

### 3.3 Assessment of risk of bias

The included studies showed varying quality levels (Supplementary S3). All studies did not show a significant risk of bias in the randomization process, deviations from intended interventions, measurement of outcomes, and selection of the reported results. However, one study (9.09%) was at high risk of bias due to missing outcome data. Four studies (40.00%) were assessed as low risk, while six (60.00%) raised some concerns.

### 3.4 Primary outcome: stroke

**FIGURE 2:**
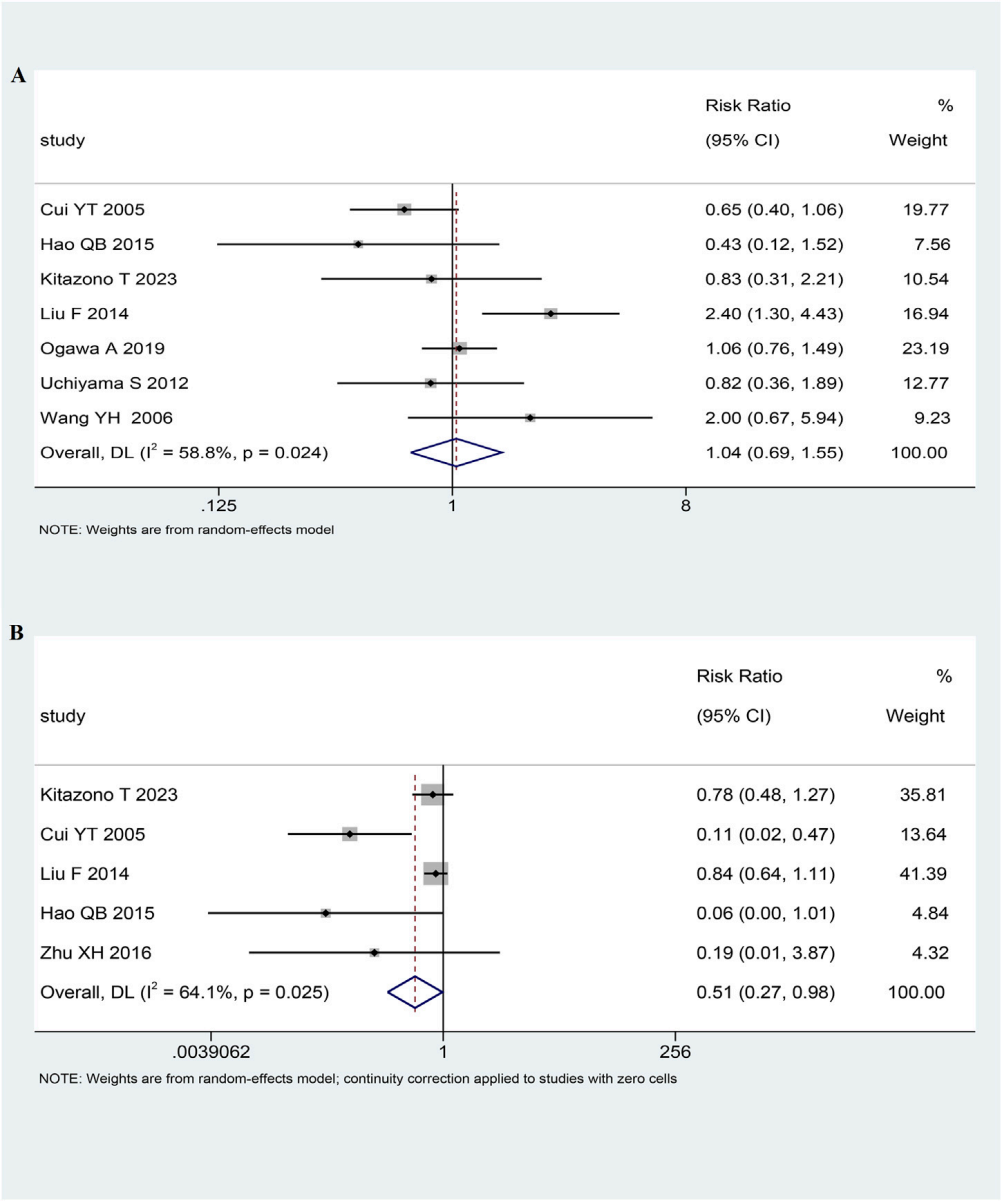
The forest plot of stroke **(A)** and bleeding **(B)**.

In seven RCTs (n = 6,854) (Kitazono et al., 2023; Ogawa et al., 2019; Uchiyama et al., 2012; Fan et al., 2014; Qingbo, 2015; Youtao, 2005; Yuehua, 2006), there were 299 (4.36%) cases of stroke. Among the 3,490 patients treated with low-dose antiplatelet drugs, 159 (4.56%) cases of stroke were reported. Similarly, among the 3,364 patients treated with antiplatelet standard-dose drugs, 140 (4.16%) cases of stroke were reported. The pooled analysis indicated that the risk of stroke was comparable between the low-dose and standard-dose antiplatelet groups (RR 1.04, 95% CI 0.69-1.55, *I* ^*2*^ = 58.8%, *p* = 0.87; Figure 2A; Table 2).

**TABLE 2:** Summary table of the results for each outcome.

Outcomes	Subgroup	Included drugs	Included study	Experimental Group (low-dose)% (n/N)	Control Group (Standard-dose)% (n/N)	*I* ^*2*^ *%*	RR	95% CI	P
MI	types of antiplatelet drugs		5	0.60 (19/3158)	0.32 (10/3123)	48.6	1.91	0.88, 4.12	0.10
		Prasugrel-low vs. Clopidogrel-standard	2	0.25 (5/2003)	0.30 (6/1974)	0.0	0.82	0.25, 2.69	0.74
		Clopidogrel-low vs. Clopidogrel/Aspirin-standard	3	1.21 (14/1155)	0.35 (4/1149)	58.1	3.60	1.16, 11.16	0.03
		Clopidogrel-low vs. Clopidogrel-standard	2	1.25 (14/1116)	0.18 (2/1104)	24.1	6.92	1.58, 30.40	0.01
		Clopidogrel-low vs. Aspirin-standard	1	0.00 (0/39)	4.44 (2/45)	-	0.23	0.01, 4.65	-
	different countries	Japan	3	0.27 (7/2,561)	0.28 (7/2,526)	0.0	0.99	0.35, 2.81	0.98
		China	2	2.01 (12/597)	0.50 (3/597)	78.2	4.17	1.14, 15.24	0.03
Stroke	types of antiplatelet drugs		7	4.56 (159/3490)	4.16 (140/3364)	58.8	1.04	0.69, 1.55	0.87
		Prasugrel-low vs. Clopidogrel-standard	2	3.79 (76/2003)	3.65 (72/1974)	0.0	1.04	0.76, 1.42	0.82
		Clopidogrel-low vs. Clopidogrel/Aspirin-standard	3	4.07 (47/1155)	2.96 (34/1149)	74.7	1.05	0.39, 2.85	0.92
		Clopidogrel-low vs. Clopidogrel-standard	2	3.94 (44/1116)	2.36 (26/1104)	75.8	1.46	0.51, 4.16	0.48
		Clopidogrel-low vs. Aspirin-standard	1	7.69 (3/39)	17.78 (8/45)	-	0.43	0.12, 1.52	0.19
		Aspirin-low vs. Aspirin-standard	2	10.84 (36/332)	14.11 (34/241)	70.6	1.02	0.35, 3.00	0.97
	different countries	Japan	3	3.36 (86/2,561)	3.33 (84/2,526)	0.0	1.01	0.75, 1.35	0.96
		China	4	7.86 (73/929)	6.68 (56/838)	78.6	1.12	0.48, 2.59	0.80
ACD	types of antiplatelet drugs		2	2.35 (8/341)	1.95 (5/256)	0.0	1.17	0.38, 3.62	0.79
		Aspirin-low vs. Aspirin-standard	1	2.65 (8/302)	1.90 (4/211)	-	1.40	0.43, 4.58	0.58
		Clopidogrel-low vs. Aspirin-standard	1	0.00 (0/39)	2.22 (1/45)	-	0.38	0.02, 9.15	-
Bleeding	types of antiplatelet drugs		5	9.82 (103/1049)	15.14 (144/951)	64.1	0.51	0.27, 0.98	0.04
		Clopidogrel-low vs. Clopidogrel/Aspirin-standard	2	13.07 (78/597)	16.92 (101/597)	69.9	0.33	0.03, 3.89	0.38
		Clopidogrel-low vs. Clopidogrel-standard	1	13.98 (78/558)	16.67 (92/552)	-	0.84	0.64, 1.11	0.22
		Clopidogrel-low vs. Aspirin-standard	1	0.00 (0/39)	20.00 (9/45)	-	0.06	0.00, 1.01	0.05
		Aspirin-low vs. Aspirin-standard	2	0.60 (2/332)	6.25 (15/240)	0.0	0.12	0.03, 0.45	0.002
		Prasugrel-low vs. Clopidogrel-standard	1	19.17 (23/120)	24.56 (28/114)	-	0.78	0.48, 1.27	0.32
	different countries	China	4	8.61 (80/929)	13.86 (116/837)	73.0	0.24	0.05, 1.10	0.07
		Japan	1	19.17 (23/120)	24.56 (28/114)	-	0.78	0.48, 1.27	0.32
Major bleeding		Prasugrel-low vs. Clopidogrel-standard	2	0.15 (3/2005)	0.20 (4/1976)	0.0	0.74	0.16, 3.30	0.69
Bleeding events leading to discontinuation		Prasugrel-low vs. Clopidogrel-standard	2	1.60 (32/2005)	1.67 (33/1976)	12.4	0.96	0.59, 1.55	0.86

-, Not applicable.

### 3.5 Primary outcome: bleeding

In five RCTs (n = 2,000) (Kitazono et al., 2023; Fan et al., 2014; Qingbo, 2015; Xinhua, 2016; Youtao, 2005), there were 247 (12.35%) cases of bleeding. Among the 1,049 patients treated with antiplatelet low-dose drugs, 103 (9.82%) cases of bleeding were reported. Similarly, among the 951 patients treated with standard-dose antiplatelet drugs, 144 (15.14%) cases of bleeding were reported. Pooled analysis showed that patients receiving low-dose antiplatelet drug treatment have a lower risk of bleeding compared to those receiving standard-dose antiplatelet drug treatment (RR 0.51, 95% CI 0.27-0.98, *I* ^*2*^ = 64.1%, *p* = 0.04; Figure 2B; Table 2).

### 3.6 Secondary outcomes

Five (n = 6,281), two (n = 597), two (n = 3,981), and two (n = 3,981) RCTs investigated MI, ACD, major bleeding, and bleeding leading to discontinuation, respectively. Compared to standard-dose antiplatelet drugs, low-dose antiplatelet drugs did not show statistically significant differences in the rates of MI, ACD, major bleeding, and bleeding leading to discontinuation. Details are shown in Table 2 and Supplementary S4.

### 3.7 Descriptive analysis

A few studies compared the efficacy and safety of low doses of different antiplatelet agents (Table 3). One study showed that patients taking 3.75 mg of prasugrel did not experience any more ischemic stroke or MI than those taking 50 mg of clopidogrel (Kitagawa et al., 2020). The incidences of MACE, MI, and stroke in the 3.75 mg prasugrel group were lower than those in the 2.5 mg prasugrel and 50 mg clopidogrel groups. The incidence rates of bleeding events in the 3.75 mg prasugrel, 2.5 mg prasugrel, and 50 mg clopidogrel groups were 31.9%, 24.7%, and 23.3%, respectively, with no significant differences in bleeding (Table 3).

**TABLE 3:** Summary table of the results for Descriptive outcome.

Outcomes	Trial registration	Randomised treatments + dose		Events % (n/N)		Hazard ratio (95% CI)
		Experimental group (low-dose)	ControlGroup (standard/low-dose)	Experimental group (low-dose)	ControlGroup (standard/low-dose)	
MACE	Kitazono et al. (2023)	Prasugrel 3.75 mg QD	Clopidogrel 75 mg QD	6.78 (8/118)	7.14 (8/112)	0.949 (0.369-2.443)^a^
	Kitagawa et al. (2020)	Prasugrel 3.75 mg QD	Clopidogrel 50 mg QD	0 (0/216)	3.59 (8/223)	-
	Kitagawa et al. (2020)	Prasugrel 2.5 mg QD	Clopidogrel 50 mg QD	3.26 (7/215)	3.59 (8/223)	0.90 (0.32–2.47)
MI	Kitagawa et al. (2020)	Prasugrel 3.75 mg QD	Clopidogrel 50 mg QD	0 (0/216)	0.9 (2/223)	-
	Kitagawa et al. (2020)	Prasugrel 2.5 mg QD	Clopidogrel 50 mg QD	0 (0/215)	0.9 (2/223)	-
STROKE	Kitagawa et al. (2020)	Prasugrel 3.75 mg QD	Clopidogrel 50 mg QD	0 (0/216)	2.69 (6/223)	-
	Kitagawa et al. (2020)	Prasugrel 2.5 mg QD	Clopidogrel 50 mg QD	3.26 (7/215)	2.69 (6/223)	1.19 (0.40–3.55)
	Zuo et al. (2017)	Clopidogrel 50 mg QD + Aspirin 100 QD	Clopidogrel 75 mg QD + Aspirin 100 mg QD	9.09 (6/66)	9.09 (6/66)	-
CVD	Zuo et al. (2017)	Clopidogrel 50 mg QD + Aspirin 100 mg QD	Clopidogrel 75 mg QD + Aspirin 100 mg QD	0 (0/66)	0 (0/66)	-
ACD	Zuo et al. (2017)	Clopidogrel 50 mg QD + Aspirin 100 mg QD	Clopidogrel 75 mg QD + Aspirin 100 mg QD	0 (0/66)	0 (0/66)	-
Bleeding	Kitagawa et al. (2020)	Prasugrel 3.75 mg QD	Clopidogrel 50 mg QD	31.94 (69/216)	23.32 (52/223)	1.40 (0.98–2.00)
	Kitagawa et al. (2020)	Prasugrel 2.5 mg QD	Clopidogrel 50 mg QD	24.65 (53/215)	23.32 (52/223)	1.04 (0.71–1.53)
Major bleeding	Kitagawa et al. (2020)	Prasugrel 3.75 mg QD	Clopidogrel 50 mg QD	0 (0/216)	0 (0/223)	-
	Kitagawa et al. (2020)	Prasugrel 2.5 mg QD	Clopidogrel 50 mg QD	0.47 (1/215)	0 (0/223)	-
Bleeding events leading to discontinuation	Kitagawa et al. (2020)	Prasugrel 3.75 mg QD	Clopidogrel 50 mg QD	2.31 (5/216)	2.24 (5/223)	1.01 (0.29–3.48)
	Kitagawa et al. (2020)	Prasugrel 2.5 mg QD	Clopidogrel 50 mg QD	0.93 (2/215)	2.24 (5/223)	0.41 (0.08–2.12)
life-threatening bleeding	Kitazono et al. (2023)	Prasugrel 3.75 mg QD	Clopidogrel 75 mg QD	0 (0/120)	0 (0/114)	-
	Kitagawa et al. (2020)	Prasugrel 3.75 mg QD	Clopidogrel 50 mg QD	1.39 (3/216)	0 (0/223)	-
	Kitagawa et al. (2020)	Prasugrel 2.5 mg QD	Clopidogrel 50 mg QD	0.47 (1/215)	0 (0/223)	-
	Ogawa et al. (2019)	Prasugrel 3.75 mg QD	Clopidogrel 75 mg QD	0.95 (18/1885)	1.24 (23/1862)	0·77 (0·41–1·42)

-, Not Applicable.

^a^ Risk Ratio (95% CI).

Studies have also compared the efficacy and safety of low-dose and standard-dose antiplatelet agents receiving dual antiplatelet therapy (Table 3). The study by Zuo FT and colleagues suggested that in Chinese stroke patients, taking aspirin (100 mg) combined with low-dose clopidogrel (50 mg) has a similar risk of stroke, ACD, and CVD compared to the combination of aspirin with the standard-dose clopidogrel (75 mg) (Zuo et al., 2017).

### 3.8 Subgroup analysis

#### 3.8.1 Subgroup analysis based on different types of antiplatelet drugs

Compared to the standard-dose clopidogrel/aspirin group, a significant increase in the risk of MI was observed in the low-dose clopidogrel group (RR 3.60, 95% CI 1.16 -11.16, *p* = 0.026). Similarly, compared to the standard-dose clopidogrel group, there was a significant increase in the risk of MI in the low-dose clopidogrel group (RR 6.92, 95% CI 1.58-30.40, *p* = 0.01; Table 2, Supplementary S5.1, 5.2). Low-dose aspirin significantly reduced the risk of bleeding compared to standard-dose aspirin (RR 0.12, 95% CI 0.03-0.45, *p* = 0.002; Table 2, Supplementary S5.1, 5.2), In terms of other outcomes, low-dose antiplatelet drugs showed similar effects compared to standard-dose antiplatelet drugs, consistent with the overall analysis results (Supplementary S5.1, 5.2). Subgroup analysis based on different countries.

Compared to the standard-dose group, the low-dose group in the Chinese population showed a significant increase in the risk of MI (RR 4.17, 95% CI 1.14-15.24, *p* = 0.03; Table 2, Supplementary S6.3). The results of other outcomes were consistent with the overall study findings (Supplementary S5.3–5.6, Supplementary S6.1–6.4).

### 3.9 Sensitivity analysis

Sensitivity analysis demonstrated that the combined effect values remained consistent before and after excluding any study for the above outcomes (Supplementary S7), suggesting that the study results were stable.

### 3.10 Publication bias analysis

**FIGURE 3:**
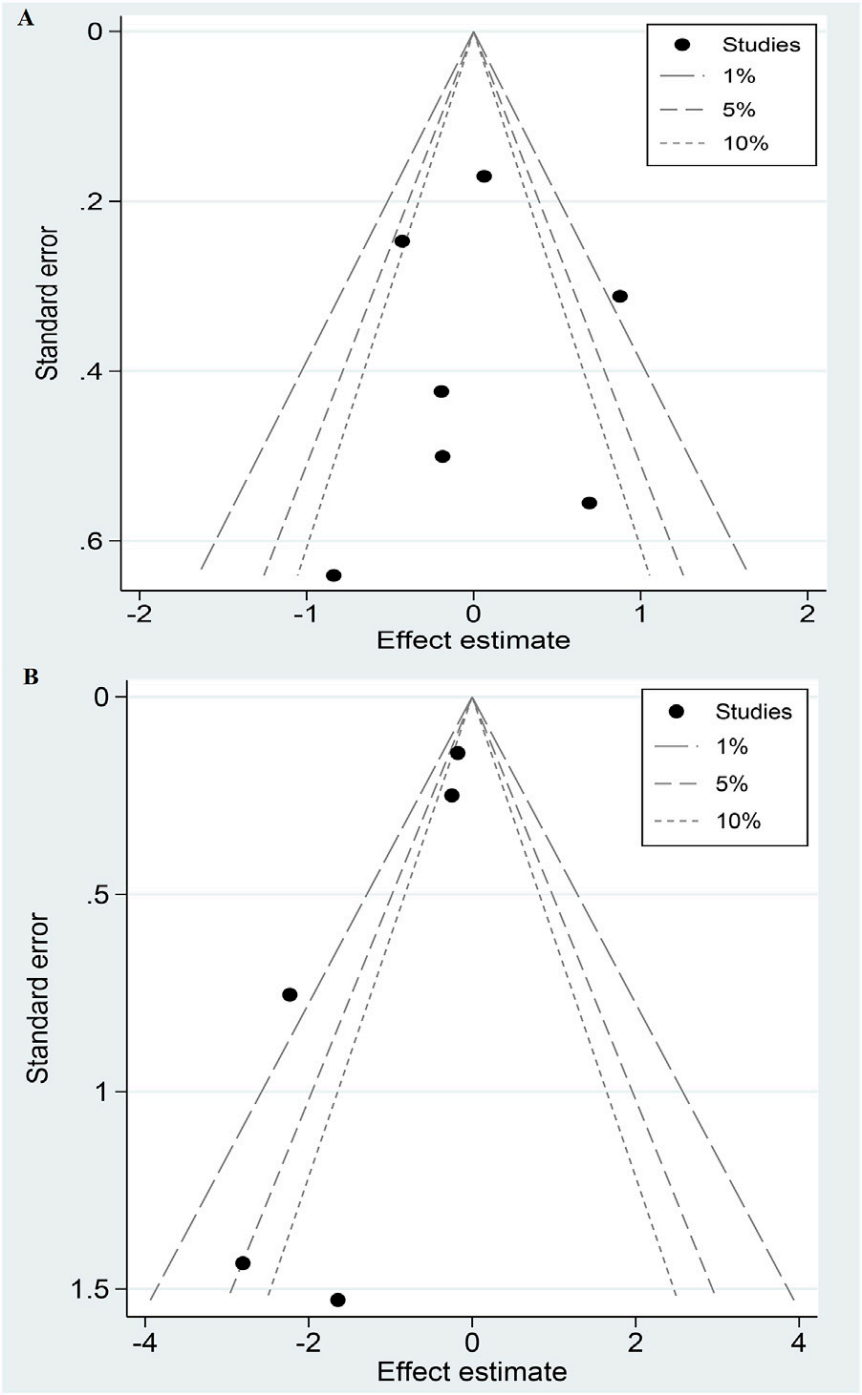
Contour-enhanced funnel plots of stroke **(A)** and bleeding **(B)**.

Contour-enhanced funnel plots suggested that bleeding may have publication bias (Figure 3), while stroke and MI showed good symmetry (Supplementary S8). Regarding Egger’s test, the P-values for stroke, MI, and bleeding were 0.95, 0.68, and 0.04, respectively, indicating that no significant publication bias for stroke and MI.

## 4 Discussion

This meta-analysis compared the efficacy and safety of low-dose versus standard-dose antiplatelet drugs in stroke patients. Ten RCTs were included, of which 2 (18.18%) were published after 2020, providing the latest evidence on the safety and efficacy of low-dose antiplatelet drugs for stroke. Low-dose antiplatelet drugs had a lower risk of bleeding compared to standard doses. Still, efficacy and safety in terms of stroke, MI, ACD, major bleeding, and bleeding leading to discontinuation were similar. The subgroup analysis revealed that, compared to the standard-dose clopidogrel/aspirin group and the standard-dose clopidogrel group, the low-dose clopidogrel group exhibited a significant increase in the risk of MI. Compared to the standard-dose group, the risk of MI in the Chinese population showed a significant increase in the low-dose clopidogrel group.

Several evidence-based pharmacological studies have evaluated the efficacy and safety of low-dose antiplatelet drugs. Among them, three (Chen et al., 2020; Hu et al., 2021; Wongsalap et al., 2022) and two (Gouya et al., 2014; McQuaid and Laine, 2006) meta-analyses have explored the efficacy and safety of low-dose and standard-dose antiplatelet drugs in patients with coronary heart disease and stroke, respectively. The range of low-dose aspirin defined in these studies was 75–325 mg/d, 150–325 mg every other day, or 75–100 mg, routine doses recommended by clinical guidelines, expert consensus, or drug labels. However, this study defined low-dose aspirin and clopidogrel as 50 mg or 25 mg. It is worth noting that the study by McQuaid and Laine (2006) included participants receiving antiplatelet therapy for primary or secondary prevention of cardiovascular disease rather than being limited to stroke patients only. In contrast, this study included only stroke patients receiving antiplatelet therapy for secondary prevention. Another difference is that this study excluded studies with a placebo control group. Due to these differences, we do not compare our results with those of the above-published studies.

A network meta-analysis (Niu et al., 2016) found that compared to standard-dose antiplatelet drugs (aspirin 75–162 mg/d, dipyridamole 75–150 mg/d, clopidogrel 75 mg/d), low-dose aspirin (30–50 mg/d) did not show significant differences in severe vascular events, prevention of recurrent stroke, and reduction of bleeding, which is consistent with our study results.

Subgroup studies suggest that, compared to standard-dose clopidogrel/aspirin, low-dose clopidogrel increases the risk of MI. Further stratification reveals a significant difference between low-dose clopidogrel and standard-dose clopidogrel in terms of increasing the risk of MI. This is the result of two pooled studies, which were mainly influenced by the research of Fan et al. (2014). Therefore, the results need to be interpreted with caution. In contrast, low-dose clopidogrel did not show statistical differences compared to standard-dose aspirin. More research is warranted, considering the relatively small sample size and the potential influence of confounding factors. The dose-response relationship shows the drug dose and effect at the individual level (Moffett et al., 2022). Based on the dose-response relationship, low-dose clopidogrel may not be sufficient to fully inhibit ischemic events. Additionally, clopidogrel is a prodrug that is primarily metabolized into its active metabolite by the liver enzyme CYP2C19 (Lee et al., 2022). CYP2C19 gene polymorphism significantly increase the risk of ischemic events such as stroke and MI (Pan et al., 2017). In Asians, the frequency of CYP2C19 gene mutations is about 2–3 times higher than in Caucasians (Huang et al., 2021). The studies included in our study did not consider the impact of genetic mutations, and therefore the results may also be influenced by CYP2C19 gene polymorphisms.

There are few retrospective studies on the efficacy and safety of low-dose antiplatelet drugs in stroke patients. One study compared the difference in neurological deficit scores and bleeding risk between low-dose clopidogrel and standard-dose clopidogrel used for 4 weeks in 114 Chinese patients with ischemic stroke (Ying et al., 2018). The results showed that the neurological deficit scores and bleeding risk were similar in both groups, which differs from our findings. Given that this study was retrospective and had a short follow-up period on the other hand. Another study compared the efficacy and safety differences between aspirin combined with low-dose clopidogrel and standard-dose clopidogrel in Chinese patients over 60 years old with intracranial atherosclerotic stenosis. The results showed that the risk of gastrointestinal bleeding in the low-dose clopidogrel group was significantly lower than that in the standard-dose clopidogrel group. Still, the composite ischemic events such as stroke and MI were equivalent between the two groups (Song et al., 2023).

This study has several strengths that enhance its reliability and relevance. First, strict definitions of low-dose antiplatelet drugs and screening of follow-up times ensure accurate results. The study focused on prasugrel, approved for percutaneous coronary intervention in Europe, the US, and Japan, demonstrating its clinical relevance. Second, the study draws on robust evidence from multicenter, double-blind studies (PRASTRO-I, PRASTRO-II) that confirm the efficacy and safety of prasugrel for non-cardioembolic stroke patients in Japan. A unique strength of this study is its inclusion of low-dose prasugrel, a dose not previously considered in meta-analyses, providing novel insights into its efficacy and safety.

This study has several limitations: (1) Only 10 studies were included, constituting a relatively small sample size; (2) The 10 articles all focused on Asian populations, mainly on Japanese and Chinese groups. Therefore, the results can only represent Asian populations and may not be applicable to other ethnic groups; (3) The diversity in the definition of bleeding standards across different studies prevented the merging of data from many studies; (4) Moderate to high heterogeneity was observed in stroke and bleeding events. There may be publication bias regarding bleeding events; (5) Subgroup analysis based on CYP2C19 genotype, renal function, and hypertension control was not feasible due to insufficient information; (6) Descriptive analysis was conducted for some studies with small sample sizes, and these findings require further confirmation.

## 5 Conclusion

In Asian stroke patients, low-dose antiplatelet drugs significantly reduced the risk of bleeding compared to standard-dose antiplatelet drugs. Still, both showed similar benefits and risks in terms of stroke, bleeding, MI, ACD, major bleeding, and bleeding leading to discontinuation. Therefore, low-dose antiplatelet drugs are recommended for Asian stroke patients. Subgroup analysis results are limited due to fewer included studies and limited sample size, and further confirmation is expected from large-scale, high-quality studies.
