# Supplementary material for: Comparing efficacy and safety of low-dose versus standard-dose antiplatelet therapy in stroke patients: a meta-analysis
Source: Front Pharmacol. 2025 Jan 6;15:1484130. doi: 10.3389/fphar.2024.1484130 (PMC11743172; doi:10.3389/fphar.2024.1484130)

**Supplementary Material**

**Content**

[Supplementary 1 Search strategy 1](#_Toc16095)

[Supplementary 2 Citations of Included Studies 10](#_Toc1960)

[Citations for the 10 eligible studies(1-10) 10](#_Toc3123)

[Supplementary 3 Risk of Bias Assessment 11](#_Toc18511)

[The results of risk of bias assessment for each study 11](#_Toc17273)

[Supplementary 4 Forest plot for meta-analysis 12](#_Toc24402)

[4.1 MI 12](#_Toc21437)

[4.2 ACD 13](#_Toc3078)

[4.3 Major bleeding 14](#_Toc22401)

[4.4 Bleeding events leading to discontinuation 15](#_Toc14652)

[Supplementary 5 Subgroup analysis by different types of antiplatelet drugs 16](#_Toc9205)

[5.1 Forest for stroke by different types of antiplatelet drugs 16](#_Toc24249)

[5.2 Forest for stroke by different types of antiplatelet drugs 17](#_Toc32144)

[5.3 Forest for bleeding by different types of antiplatelet drugs 17](#_Toc13269)

[5.4 Forest for bleeding by different types of antiplatelet drugs 19](#_Toc6169)

[5.5 Forest for MI by different types of antiplatelet drugs 20](#_Toc22463)

[5.6 Forest for MI by different types of antiplatelet drugs 21](#_Toc3688)

[5.7 Forest for ACD by different types of antiplatelet drugs 22](#_Toc6351)

[Supplementary 6 Subgroup analysis by different countries 23](#_Toc229)

[6.1 Forest for stroke by different countries 23](#_Toc15218)

[6.2 Forest for bleeding by different countries 24](#_Toc22858)

[6.3 Forest for MI by different countries 25](#_Toc25978)

[Supplementary 7 Results of sensitive analysis 26](#_Toc25909)

[7.1 Sensitive analysis of stroke 26](#_Toc14544)

[7.2 Sensitive analysis of bleeding 27](#_Toc18061)

[7.3 Sensitive analysis of MI 28](#_Toc16605)

[7.4 Sensitive analysis of ACD 29](#_Toc28615)

[Supplementary 8 Confunnel plots 30](#_Toc12331)

[Confunnel plotsfor MI 30](#_Toc21384)

#

# Abbreviations

ACD=all-cause death

ACS=acute coronary syndrome

BID=Bis in die

CNKI=China National Knowledge Infrastructure

MI=myocardial infarction

NA=Not applicable

QD=Quaque die

TID=Ter in die

UA=unstable angina

# Supplementary 1 Search strategy

Pubmed, Embase , the Cochrane Controlled Register of Trials, ClinicalTrials.gov, CNKI (China National Knowledge Infrastructure) and Wanfang Data Knowledge Service Platform from database inception to Mar. 27, 2024.

The detailed search strategy for each database was presented below:

**Pubmed**

**Participation(****Stroke) related**

#1"Stroke"[MeSH Terms]

#2"Ischemic stroke"[MeSH Terms]

#3"Strokes"[Title/Abstract]

#4"Cerebrovascular Accident"[Title/Abstract]

#5"Cerebrovascular Accidents"[Title/Abstract]

#6"Cerebrovascular Stroke"[Title/Abstract]

#7"Cerebrovascular Strokes"[Title/Abstract]

#8"Stroke, Cerebrovascular"[Title/Abstract]

#9"Apoplexy"[Title/Abstract]

#10"Cerebral Stroke"[Title/Abstract]

#11"Cerebral Strokes"[Title/Abstract]

#12"Stroke, Cerebral"[Title/Abstract]

#13"Stroke, Acute"[Title/Abstract]

#14"Acute Stroke"[Title/Abstract]

#15"Acute Strokes"[Title/Abstract]

#16"Acute Cerebrovascular Accident"[Title/Abstract]

#17"Acute Cerebrovascular Accidents"[Title/Abstract]

#18"cerebral ischemic stroke"[Title/Abstract]

#19"Ischaemic Stroke"[Title/Abstract]

#20"Acute Ischemic Stroke"[Title/Abstract]

#21"Ischemic Strokes"[Title/Abstract]

#22"Stroke, Ischemic"[Title/Abstract]

#23"Ischaemic Stroke"[Title/Abstract]

#24"Ischaemic Strokes"[Title/Abstract]

#25"Stroke, Ischaemic"[Title/Abstract]

#26"Cryptogenic Ischemic Stroke"[Title/Abstract]

#27"Cryptogenic Stroke"[Title/Abstract]

#28"Cryptogenic Strokes"[Title/Abstract]

#29"Wake-up Stroke"[Title/Abstract]

#30"Wake up Stroke"[Title/Abstract]

#31"Wake-up Strokes"[Title/Abstract]

#32"Acute Ischemic Strokes"[Title/Abstract]

#33"Ischemic Stroke, Acute"[Title/Abstract]

#34 OR #1-33

**Intervention (Platelet Aggregation Inhibitors) related**

#35"Platelet Aggregation Inhibitors"[MeSH Terms]

#36"Aspirin"[MeSH Terms]

#37"Clopidogrel"[MeSH Terms]

#38"ticagrelor"[MeSH Terms]

#39"Purinergic P2Y Receptor Antagonists"[MeSH Terms]

#40"Cilostazol"[MeSH Terms]

#41"Dipyridamole"[MeSH Terms]

#42"Prasugrel Hydrochloride"[MeSH Terms]

#43"Blood Platelet Aggregation Inhibitor"[Title/Abstract]

#44"Blood Platelet Aggregation Inhibitors"[Title/Abstract]

#45"Platelet Antiaggregants"[Title/Abstract]

#46"Platelet Inhibitors"[Title/Abstract]

#47"Platelet Inhibitor"[Title/Abstract]

#48"Antiplatelet Agents"[Title/Abstract]

#49"Antiplatelet Agent"[Title/Abstract]

#50"Antiplatelet Drug"[Title/Abstract]

#51"Antiplatelet Drugs"[Title/Abstract]

#52"Platelet Antagonists"[Title/Abstract]

#53"Acetylsalicylic Acid"[Title/Abstract]

#54"ASA"[Title/Abstract]

#55"Iscover"[Title/Abstract]

#56"PCR 4099"[Title/Abstract]

#57"SR 25989"[Title/Abstract]

#58"Plavix"[Title/Abstract]

#59"Clopidogrel Bisulfate"[Title/Abstract]

#60"thromboxane A2 antagonist"[Title/Abstract]

#61"Indobufen"[Title/Abstract]

#62"Ibustrin"[Title/Abstract]

#63"K 3920"[Title/Abstract]

#64"Brilique"[Title/Abstract]

#65"AZD 6140"[Title/Abstract]

#66"AZD6140"[Title/Abstract]

#67"AZD-6140"[Title/Abstract]

#68"Brilinta"[Title/Abstract]

#69"Purinergic P2Y12 Receptor Antagonists"[Title/Abstract]

#70"P2Y12 Purinoceptor Antagonist"[Title/Abstract]

#71"P2Y12 Receptor Antagonists"[Title/Abstract]

#72"P2Y12 Receptor Antagonist"[Title/Abstract]

#73"P2Y12 Purinoceptor Antagonists"[Title/Abstract]

#74"OPC 13013"[Title/Abstract]

#75"OPC-13013"[Title/Abstract]

#76"Pletal"[Title/Abstract]

#77"Dipyramidole"[Title/Abstract]

#78"sarpogrelate"[Title/Abstract]

#79"Persantine"[Title/Abstract]

#80"Persantin"[Title/Abstract]

#81"MCI 9042"[Title/Abstract]

#82"MCI-9042"[Title/Abstract]

#83"CS-747"[Title/Abstract]

#84"CS747"[Title/Abstract]

#85"Prasugrel"[Title/Abstract]

#86"LY 640315"[Title/Abstract]

#87 OR #35-86

**Low dose related**

#88"low dose"[All Fields]

#89"low-dose"[All Fields]

#90"50mg"[All Fields]

#91"25mg"[All Fields]

#92"50 mg twice daily"[All Fields]

#93"5mg"[All Fields]

#94"3.75mg"[All Fields]

#95"60mg"[All Fields]

#96"45 mg bid"[All Fields]

#97"45 mg twice daily"[All Fields]

#98OR #111-120

#99#34AND #87 AND #98

**Studies (randomized controlled trial) related**

**#99**

(randomized controlled trial[pt] OR controlled clinical trial[pt] OR randomized[tiab] OR placebo[tiab] OR drug therapy[sh] OR randomly[tiab] OR trial[tiab] OR groups[tiab]) NOT (animals[mh] NOT humans[mh])

#100#34AND #87 AND #98 AND #99

**EMBASE**

**Participation (Stroke) related**

#1 'cerebrovascular accident'/de

#2 'Ischemic stroke'/de

#3'Strokes':ti,ab,kw

#4'Cerebrovascular Accident':ti,ab,kw

#5'Cerebrovascular Accidents':ti,ab,kw

#6'Cerebrovascular Stroke':ti,ab,kw

#7'Stroke, Cerebrovascular':ti,ab,kw

#8'Apoplexy':ti,ab,kw

#9'Cerebral Stroke':ti,ab,kw

#10'Cerebral Strokes':ti,ab,kw

#11'Stroke, Cerebral':ti,ab,kw

#12'Stroke, Acute':ti,ab,kw

#13'Acute Stroke':ti,ab,kw

#14'Acute Strokes':ti,ab,kw

#15'Acute Cerebrovascular Accident':ti,ab,kw

#16'cerebral ischemic stroke':ti,ab,kw

#17'Ischaemic Stroke':ti,ab,kw

#18'Acute Ischemic Stroke':ti,ab,kw

#19'Ischemic Strokes':ti,ab,kw

#20'Stroke, Ischemic':ti,ab,kw

#21'Ischaemic Stroke':ti,ab,kw

#22'Ischaemic Strokes':ti,ab,kw

#23'Stroke, Ischaemic':ti,ab,kw

#24'Cryptogenic Ischemic Stroke':ti,ab,kw

#25'Cryptogenic Stroke':ti,ab,kw

#26'Cryptogenic Strokes':ti,ab,kw

#27'Acute Ischemic Strokes':ti,ab,kw

#28 OR #1-27

**Intervention (Platelet Aggregation Inhibitors) related**

#29 'antithrombocytic agent'/de

#30 'acetylsalicylic acid'/de

#31 'clopidogrel'/de

#32 'ticagrelor'/de

#33 'purinergic p2y receptor antagonists'/de

#34 'cilostazol'/de

#35 'dipyridamole'/de

#36 'prasugrel'/de

#37 'blood platelet aggregation inhibitor':ti,ab,kw

#38 'blood platelet aggregation inhibitors':ti,ab,kw

#39 'platelet antiaggregants':ti,ab,kw

#40 'platelet inhibitors':ti,ab,kw

#41 'platelet inhibitor':ti,ab,kw

#42 'antiplatelet agents':ti,ab,kw

#43 'antiplatelet agent':ti,ab,kw

#44 'antiplatelet drug':ti,ab,kw

#45 'antiplatelet drugs':ti,ab,kw

#46 'platelet antagonists':ti,ab,kw

#47 'acetylsalicylic acid':ti,ab,kw

#48 'iscover':ti,ab,kw

#49 'pcr 4099':ti,ab,kw

#50 'sr 25989':ti,ab,kw

#51 'plavix':ti,ab,kw

#52 'clopidogrel bisulfate':ti,ab,kw

#53 'thromboxane a2 antagonist':ti,ab,kw

#54 'indobufen':ti,ab,kw

#55 'ibustrin':ti,ab,kw

#56 'k 3920':ti,ab,kw

#57 'brilique':ti,ab,kw

#58 'azd 6140':ti,ab,kw

#59 'azd6140':ti,ab,kw

#60 'azd-6140':ti,ab,kw

#61 'brilinta':ti,ab,kw

#62 'purinergic p2y12 receptor antagonists':ti,ab,kw

#63 'p2y12 purinoceptor antagonist':ti,ab,kw

#64 'p2y12 receptor antagonists':ti,ab,kw

#65 'p2y12 receptor antagonist':ti,ab,kw

#66 'p2y12 purinoceptor antagonists':ti,ab,kw

#67 'opc 13013':ti,ab,kw

#68 'opc-13013':ti,ab,kw

#69'pletal':ti,ab,kw

#70 'dipyramidole':ti,ab,kw

#71 'sarpogrelate':ti,ab,kw

#72 'persantine':ti,ab,kw

#73 'persantin':ti,ab,kw

#74 'mci 9042':ti,ab,kw

#75 'mci-9042':ti,ab,kw

#76 'cs-747':ti,ab,kw

#77 'cs747':ti,ab,kw

#78 'prasugrel':ti,ab,kw

#79 'ly 640315':ti,ab,kw

#80 OR #29-#79

**Low dose related**

#81'low drug dose'/de

#82'low dose'all fields

#83'low-dose'all fields

#84 OR #81-#83

**Studies (randomized controlled trial) related**

**#85**

'crossover procedure':de OR 'double-blind procedure':de OR 'randomized controlled trial':de OR 'single-blind procedure':de OR (random* OR factorial* OR crossover* OR cross NEXT/1 over* OR placebo* OR doubl* NEAR/1 blind* OR singl* NEAR/1 blind* OR assign* OR allocat* OR volunteer*):de,ab,ti

#86 #28 AND #80 AND #84 AND #85

**Cochrane**

**Participation(Stroke) related**

#1MeSH descriptor: [Stroke]this term only

#2MeSH descriptor: [Ischemic stroke]this term only

#3("Strokes"):ti,ab,kw

#4("Cerebrovascular Accident"):ti,ab,kw

#5("Cerebrovascular Accidents"):ti,ab,kw

#6("Cerebrovascular Stroke"):ti,ab,kw

#7("Cerebrovascular Strokes"):ti,ab,kw

#8("Stroke, Cerebrovascular"):ti,ab,kw

#9("Apoplexy"):ti,ab,kw

#10("Cerebral Stroke"):ti,ab,kw

#11("Cerebral Strokes"):ti,ab,kw

#12("Stroke, Cerebral"):ti,ab,kw

#13("Stroke, Acute"):ti,ab,kw

#14("Acute Stroke"):ti,ab,kw

#15("Acute Strokes"):ti,ab,kw

#16("Acute Cerebrovascular Accident"):ti,ab,kw

#17("Acute Cerebrovascular Accidents"):ti,ab,kw

#18("cerebral ischemic stroke"):ti,ab,kw

#19("Ischaemic Stroke"):ti,ab,kw

#20("Acute Ischemic Stroke"):ti,ab,kw

#21("Ischemic Strokes"):ti,ab,kw

#22("Stroke, Ischemic"):ti,ab,kw

#23("Ischaemic Stroke"):ti,ab,kw

#24("Ischaemic Strokes"):ti,ab,kw

#25("Stroke, Ischaemic"):ti,ab,kw

#26("Cryptogenic Ischemic Stroke"):ti,ab,kw

#27("Cryptogenic Stroke"):ti,ab,kw

#28("Cryptogenic Strokes"):ti,ab,kw

#29("Wake-up Stroke"):ti,ab,kw

#30("Wake up Stroke"):ti,ab,kw

#31("Wake-up Strokes"):ti,ab,kw

#32("Acute Ischemic Strokes"):ti,ab,kw

#33("Ischemic Stroke, Acute"):ti,ab,kw

#34 OR #1-33

**Intervention (Platelet Aggregation Inhibitors) related**

#35MeSH descriptor: [Platelet Aggregation Inhibitors]this term only

#36MeSH descriptor: [Aspirin]this term only

#37MeSH descriptor: [Clopidogrel]this term only

#38MeSH descriptor: [ticagrelor]this term only

#39MeSH descriptor: [Purinergic P2Y Receptor Antagonists]this term only

#40MeSH descriptor: [Cilostazol]this term only

#41MeSH descriptor: [Dipyridamole]this term only

#42MeSH descriptor: [Prasugrel Hydrochloride]this term only

#43("Blood Platelet Aggregation Inhibitor"):ti,ab,kw

#44("Blood Platelet Aggregation Inhibitors"):ti,ab,kw

#45("Platelet Antiaggregants"):ti,ab,kw

#46("Platelet Inhibitors"):ti,ab,kw

#47("Platelet Inhibitor"):ti,ab,kw

#48("Antiplatelet Agents"):ti,ab,kw

#49("Antiplatelet Agent"):ti,ab,kw

#50("Antiplatelet Drug"):ti,ab,kw

#51("Antiplatelet Drugs"):ti,ab,kw

#52("Platelet Antagonists"):ti,ab,kw

#53("Acetylsalicylic Acid"):ti,ab,kw

#54("ASA"):ti,ab,kw

#55("Iscover"):ti,ab,kw

#56("PCR 4099"):ti,ab,kw

#57("SR 25989"):ti,ab,kw

#58("Plavix"):ti,ab,kw

#59("Clopidogrel Bisulfate"):ti,ab,kw

#60("thromboxane A2 antagonist"):ti,ab,kw

#61("Indobufen"):ti,ab,kw

#62("Ibustrin"):ti,ab,kw

#63("K 3920"):ti,ab,kw

#64("Brilique"):ti,ab,kw

#65("AZD 6140"):ti,ab,kw

#66("AZD6140"):ti,ab,kw

#67("AZD-6140"):ti,ab,kw

#68("Brilinta"):ti,ab,kw

#69("Purinergic P2Y12 Receptor Antagonists"):ti,ab,kw

#70("P2Y12 Purinoceptor Antagonist"):ti,ab,kw

#71("P2Y12 Receptor Antagonists"):ti,ab,kw

#72("P2Y12 Receptor Antagonist"):ti,ab,kw

#73("P2Y12 Purinoceptor Antagonists"):ti,ab,kw

#74("OPC 13013"):ti,ab,kw

#75("OPC-13013"):ti,ab,kw

#76("Pletal"):ti,ab,kw

#77("Dipyramidole"):ti,ab,kw

#78("sarpogrelate"):ti,ab,kw

#79("Persantine"):ti,ab,kw

#80("Persantin"):ti,ab,kw

#81("MCI 9042"):ti,ab,kw

#82 ("MCI-9042"):ti,ab,kw

#83("CS-747"):ti,ab,kw

#84("CS747"):ti,ab,kw

#85("Prasugrel"):ti,ab,kw

#86("LY 640315"):ti,ab,kw

#87 OR #35-86

**Low dose related**

#88"low dose"[All Text]

#89"low-dose"[All Text]

#90"50mg"[All Text]

#91"25mg"[All Text]

#92"50 mg twice daily"[All Text]

#93"5mg"[All Text]

#94"3.75mg"[All Text]

#95"60mg"[All Text]

#96"45 mg bid"[All Text]

#97"45 mg twice daily"[All Text]

#98OR #111-120

#99#34AND #87 AND #98

**Studies (randomized controlled trial) related**

#100 MeSH descriptor: [Randomized Controlled Trials as Topic] this term only

#101 ("randomized controlled trial"):ti,ab,kw

#102 ("randomized control trial"):ti,ab,kw

#103 ("randomized controlled trials"):ti,ab,kw

#104 ("rct"):ti,ab,kw

#105 ("rcts"):ti,ab,kw

#106 ("randomized"):ti,ab,kw

#107 ("placebo"):ti,ab,kw

#108 ("randomly"):ti,ab,kw

#109 OR #100-108

#110 #99 AND #109

**CNKI, Wanfang Data Knowledge Service Platform**

The following are the corresponding English translations of the Chinese subject terms：

**Participants (stroke) related**

Stroke

Ischemic stroke

Cerebrovascular Accident

Cerebrovascular Stroke

Cerebral ischemic stroke

Cerebral apoplexy

Acute stroke

Acute cerebrovascular accident

Acute ischemic stroke

Cryptogenic stroke

**Intervention (Platelet Aggregation Inhibitors) related**

Aspirin

Clopidogrel

ticagrelor

Cilostazol

Prasugrel

Indobufen

Antiplatelet Agents

Dipyridamole

Blood Platelet Aggregation Inhibitor

Platelet Aggregation Inhibitors

**Studies (randomized controlled trial) related**

randomized controlled trial

RCT

RCTs

**Low dose related**

Low dose

# Supplementary 2 Citations of Included Studies

## Citations for the 10 eligible studies(1-10)

1. Kitazono T, Kamouchi M, Matsumaru Y et al. Efficacy and Safety of Prasugrel vs Clopidogrel in Thrombotic Stroke Patients With Risk Factors for Ischemic Stroke Recurrence: A Double-blind, Phase III Study (PRASTRO-III). Journal of atherosclerosis and thrombosis 2023;30:222-236.

2. Kitagawa K, Toyoda K, Kitazono T et al. Safety and Efficacy of Prasugrel in Elderly/Low Body Weight Japanese Patients with Ischemic Stroke: Randomized PRASTRO-II. Cerebrovascular diseases (Basel, Switzerland) 2020;49:152-159.

3. Ogawa A, Toyoda K, Kitagawa K et al. Comparison of prasugrel and clopidogrel in patients with non-cardioembolic ischaemic stroke: a phase 3, randomised, non-inferiority trial (PRASTRO-I). The Lancet Neurology 2019;18:238-247.

4. Zuo FT, Liu H, Wu HJ, Su N, Liu JQ, Dong AQ. The effectiveness and safety of dual antiplatelet therapy in ischemic cerebrovascular disease with intracranial and extracranial arteriostenosis in Chinese patients: A randomized and controlled trail. Medicine 2017;96:e5497.

5. Uchiyama S, Tanahashi N, Minematsu K. Clopidogrel two doses comparative 1-year assessment of safety and efficacy (COMPASS) study in Japanese patients with ischemic stroke. Cerebrovascular diseases (Basel, Switzerland) 2012;34:229-39.

6. Youtao C: Aspirin in the prevention of isdmemic cerebrovascular disease. Anhui Medical 2005, 26(2):96-98.

7. Xinhua Z: Clinical observation of aspirin in treatment of acute cerebral infarction after cerebral hemorrhage. Guangxi University of Chinese Medicine 2017.

8. Qingbo H: Clinical Observation Of Clopidogrel And Aspirin In Treatment And Prevention Of Cerebral Infarction. Jilin University 2015.

9. Liu Fan CT, Liu Xuejuan, et al: Comparison of Efficacy and Safety of Different Doses of Clopidogrel Sulfate in the Treatment of Ischemic Stroke. Herald of Medicine 2014, 33(2):194-197.

10. YueHua W: Comparison of the Efficacy of Enteric-Coated Aspirin in Preventing and Treating Stroke in High-Risk Patients. Practical Journal of Cardiac Cerebral Pneumal 2006, 14(1):60-61.

# Supplementary 3 Risk of Bias Assessment

## The results of risk of bias assessment for each study

| Study | Randomization process | Deviations from intended interventions | Missing outcome data | Measurement of the outcome | Selection of the reported result | Overall |
| --- | --- | --- | --- | --- | --- | --- |
| Kitazono T 2023 | 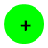 | 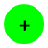 | 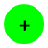 | 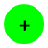 | 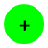 | 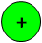 |
| Ogawa A 2019 | 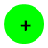 | 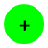 | 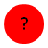 | 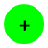 | 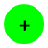 | 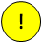 |
| Kitagawa K 2020 | 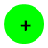 | 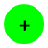 | 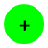 | 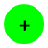 | 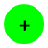 | 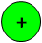 |
| Zuo FT 2017 | 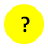 | 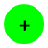 | 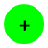 | 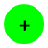 | 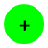 | 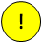 |
| Uchiyama S 2012 | 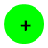 | 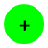 | 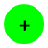 | 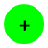 | 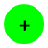 | 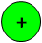 |
| Cui YT 2005 | 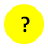 | 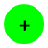 | 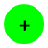 | 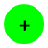 | 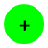 | 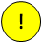 |
| Wang YH 2006 | 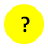 | 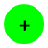 | 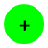 | 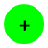 | 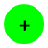 | 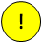 |
| Liu F 2014 | 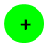 | 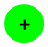 | 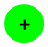 | 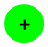 | 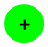 | 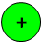 |
| Hao QB 2015 | 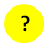 | 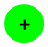 | 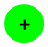 | 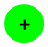 | 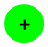 | 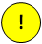 |
| Zhu XH 2016 | 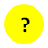 | 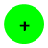 | 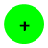 | 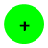 | 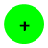 | 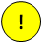 |


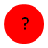

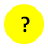

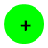


: Low risk : Some concerns : High risk


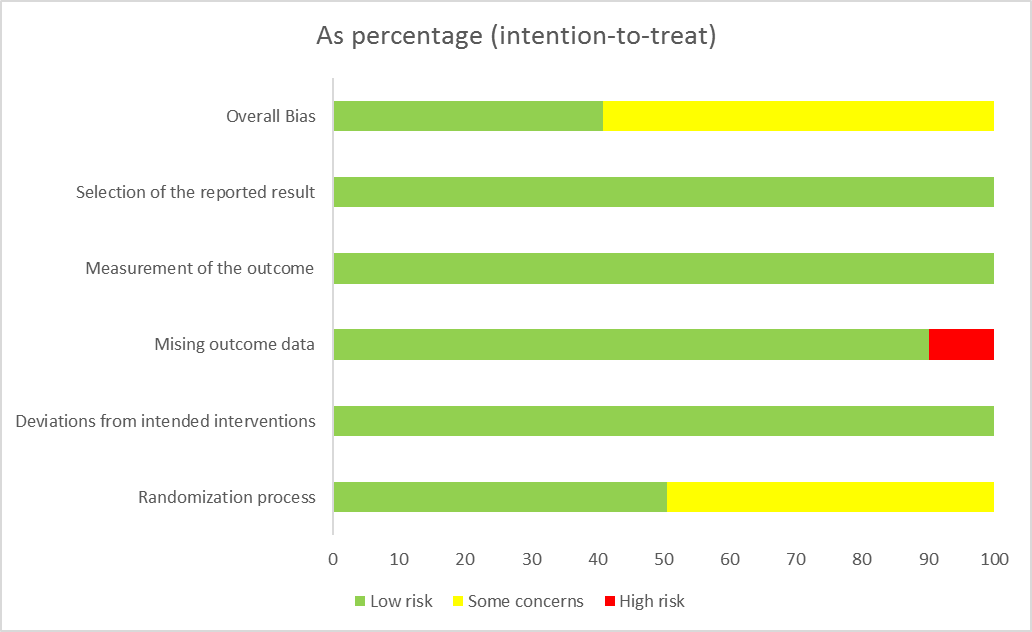


# Supplementary 4 Forest plot for meta-analysis

## 4.1 MI


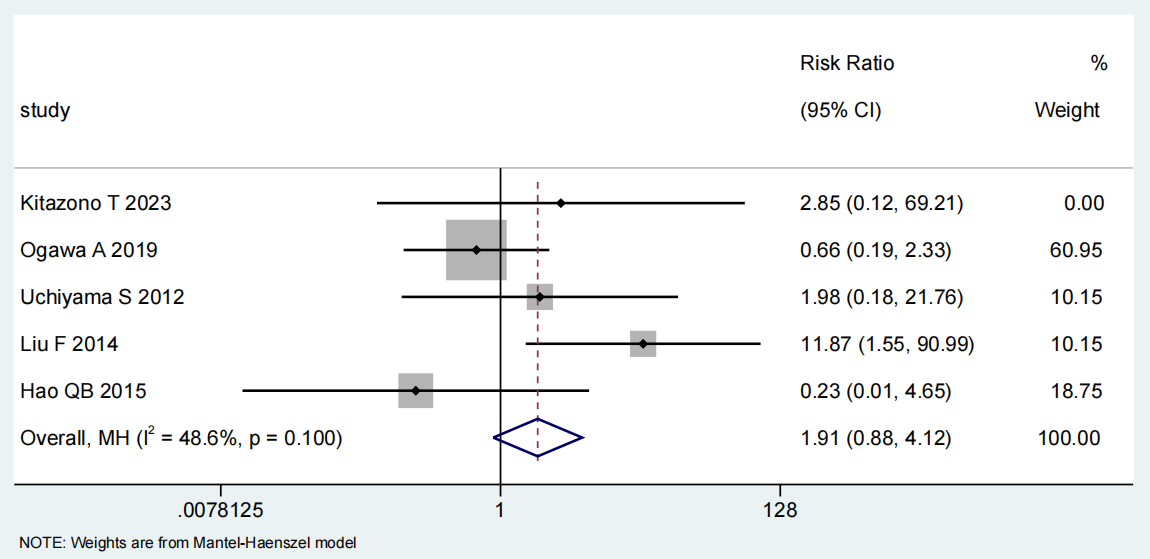


#

## 4.2 ACD


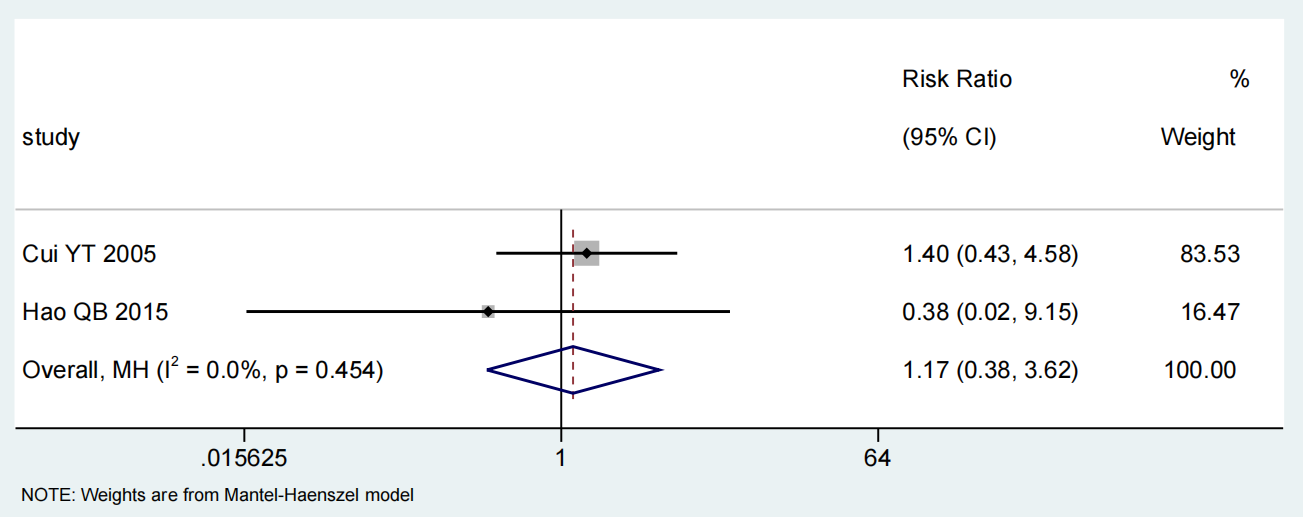


#

## 4.3 Major bleeding


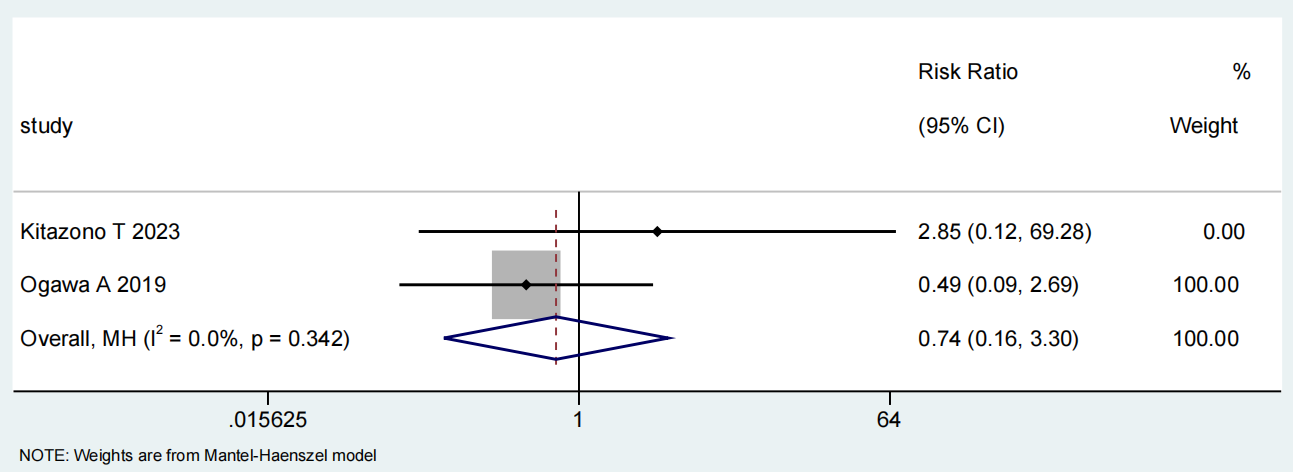


## 4.4 Bleeding events leading to discontinuation


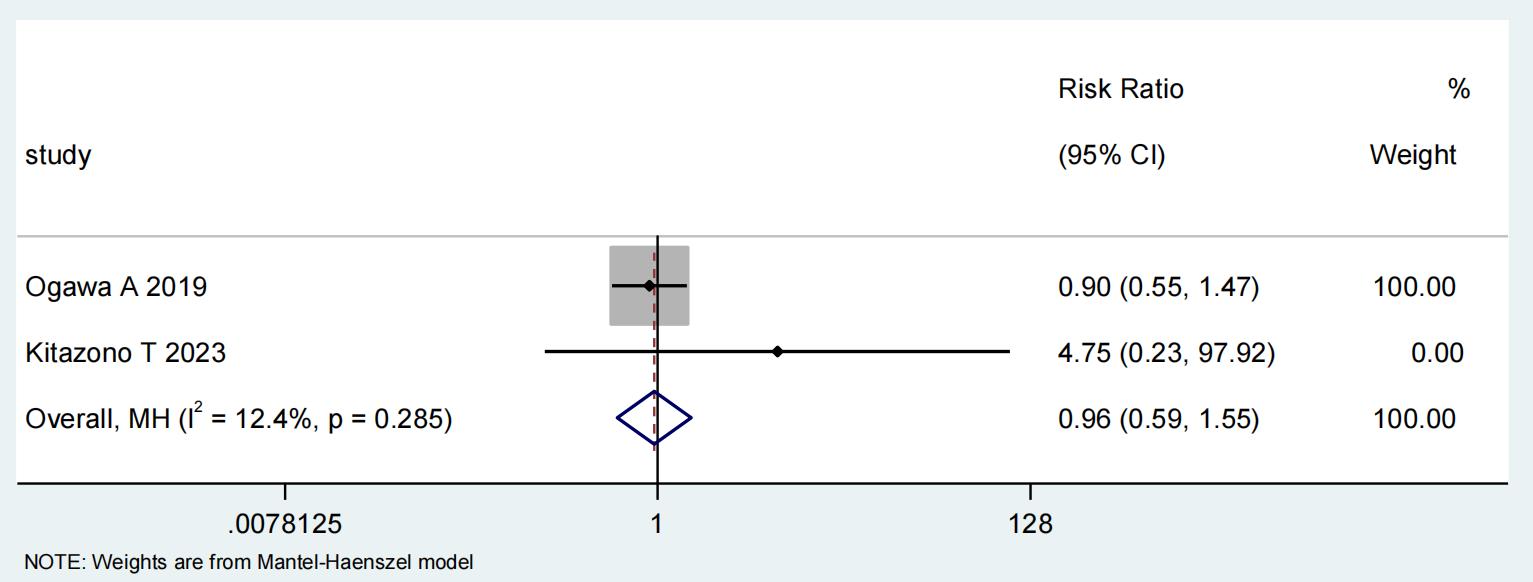


# Supplementary 5 Subgroup analysis by different types of antiplatelet drugs

## 5.1 Forest for stroke by different types of antiplatelet drugs


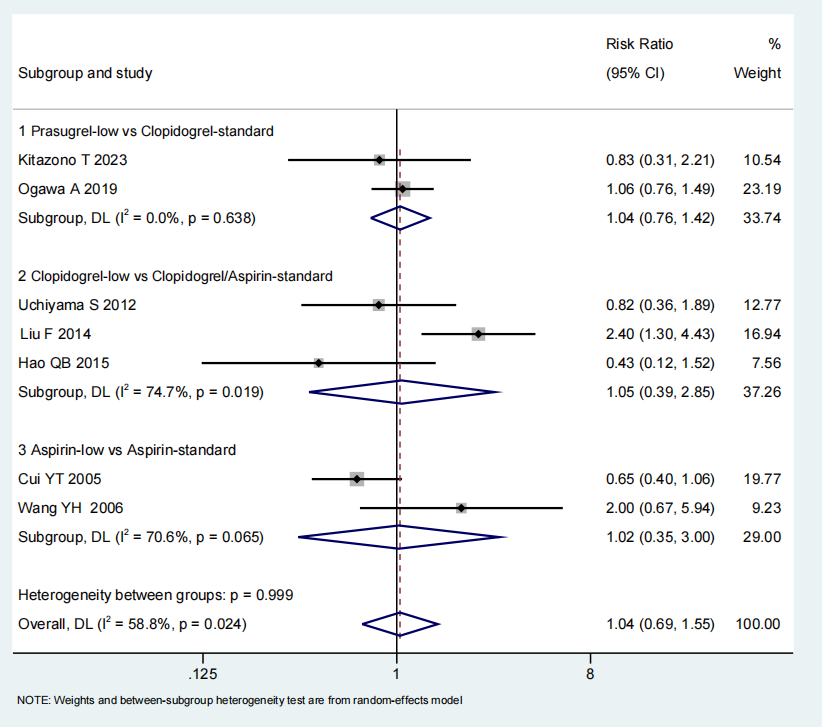


## 5.2 Forest for stroke by different types of antiplatelet drugs

##
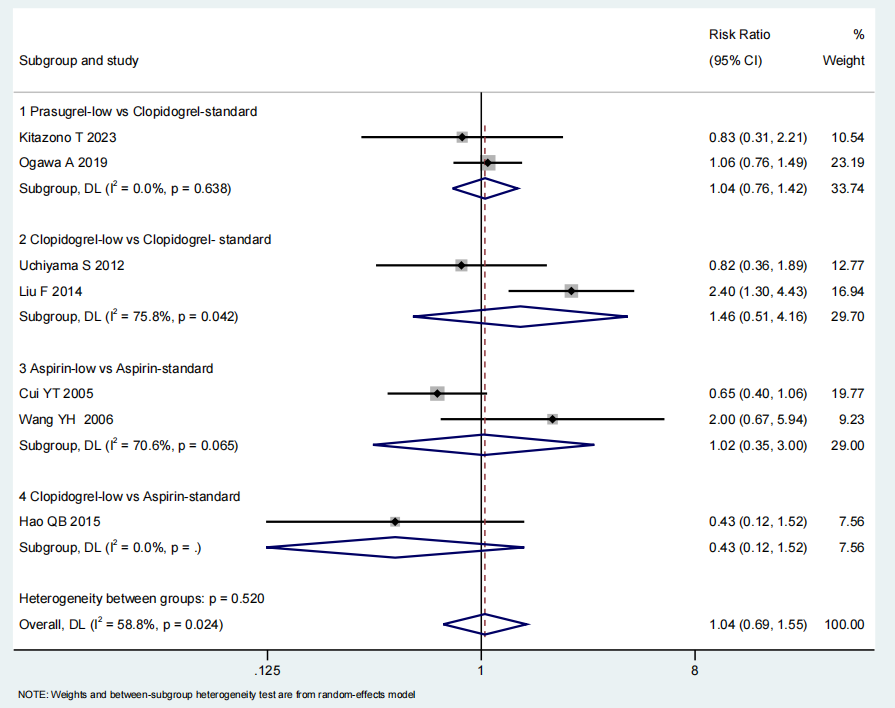
5.3 Forest for bleeding by different types of antiplatelet drugs


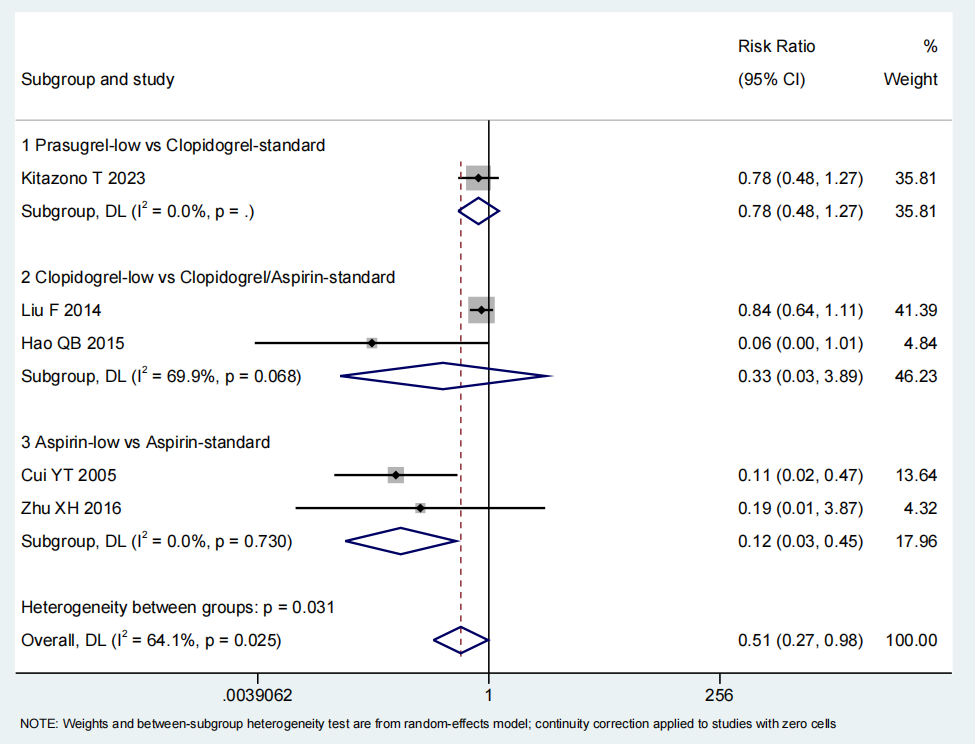


#

## 5.4 Forest for bleeding by different types of antiplatelet drugs


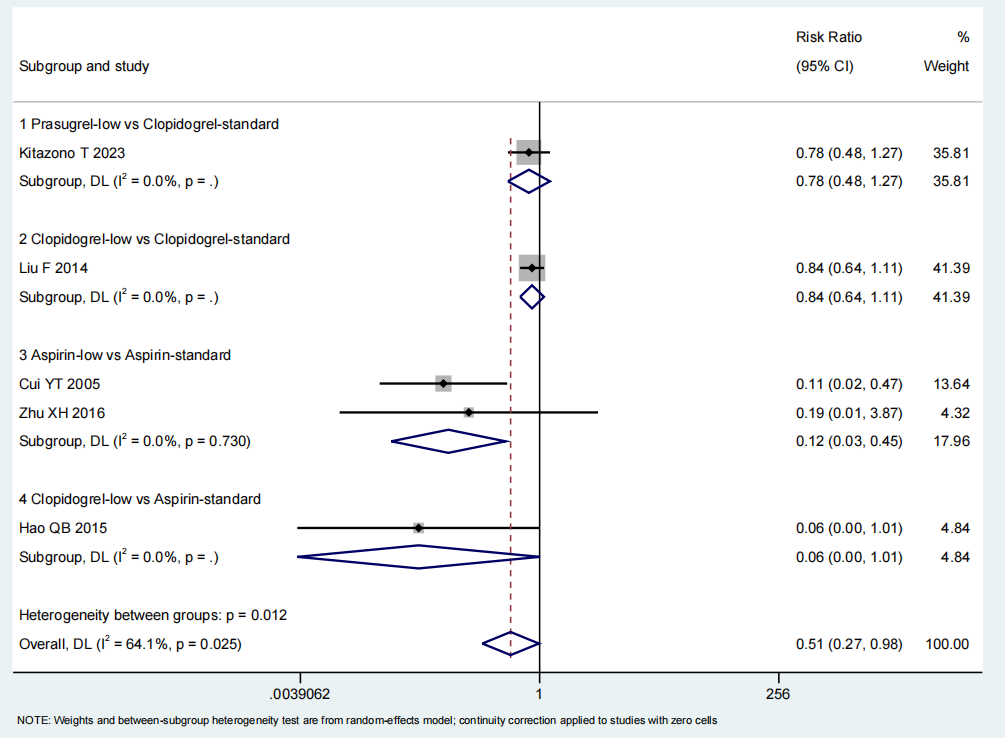


## 5.5 Forest for MI by different types of antiplatelet drugs


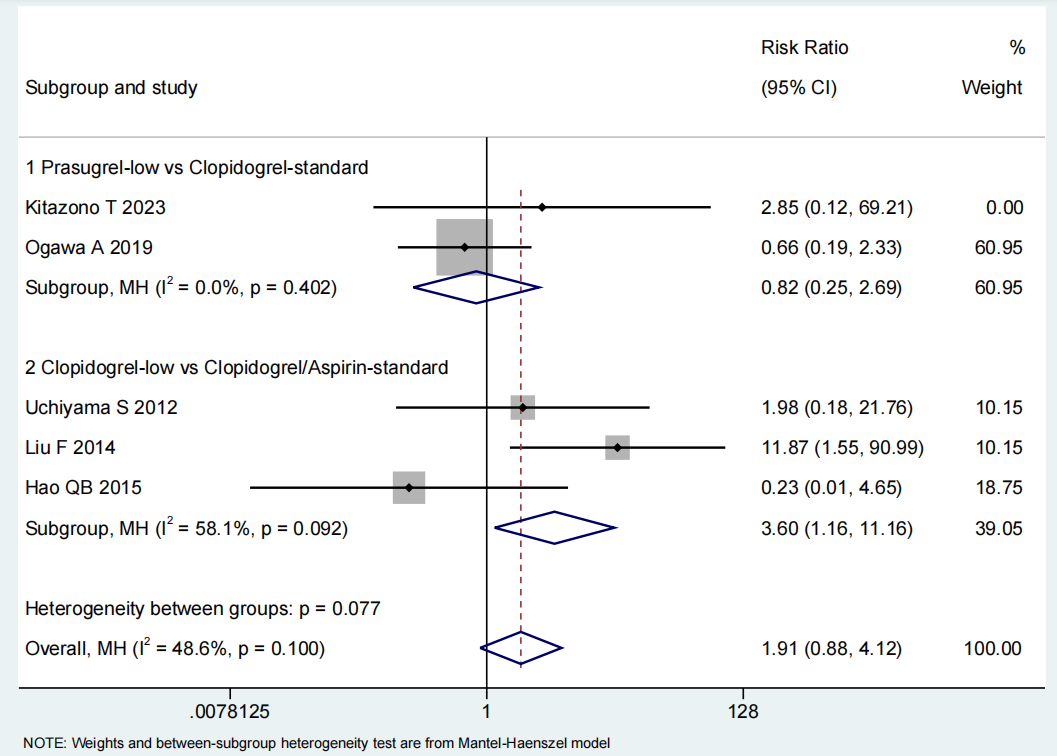


## 5.6 Forest for MI by different types of antiplatelet drugs


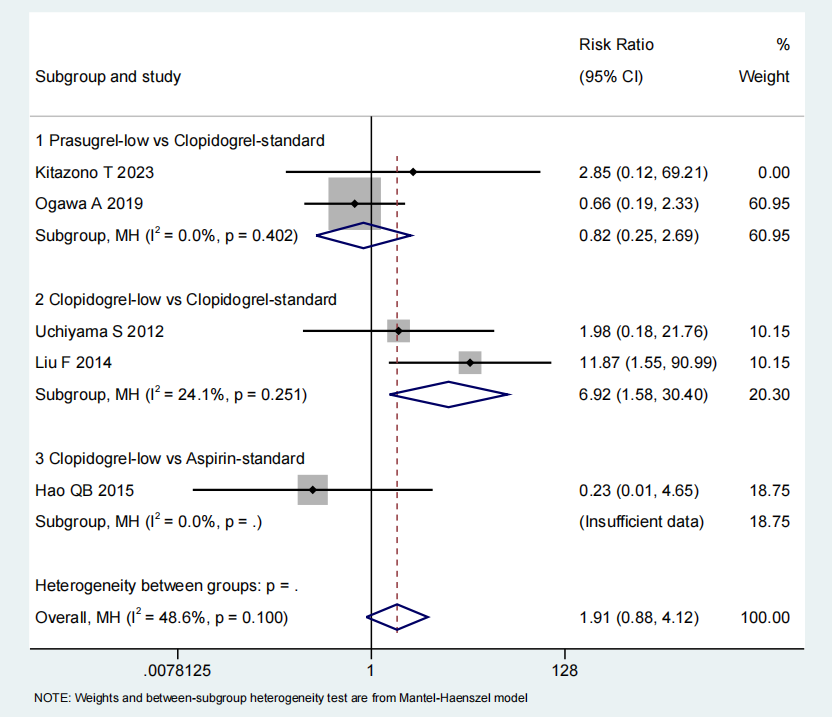


## 5.7 Forest for ACD by different types of antiplatelet drugs


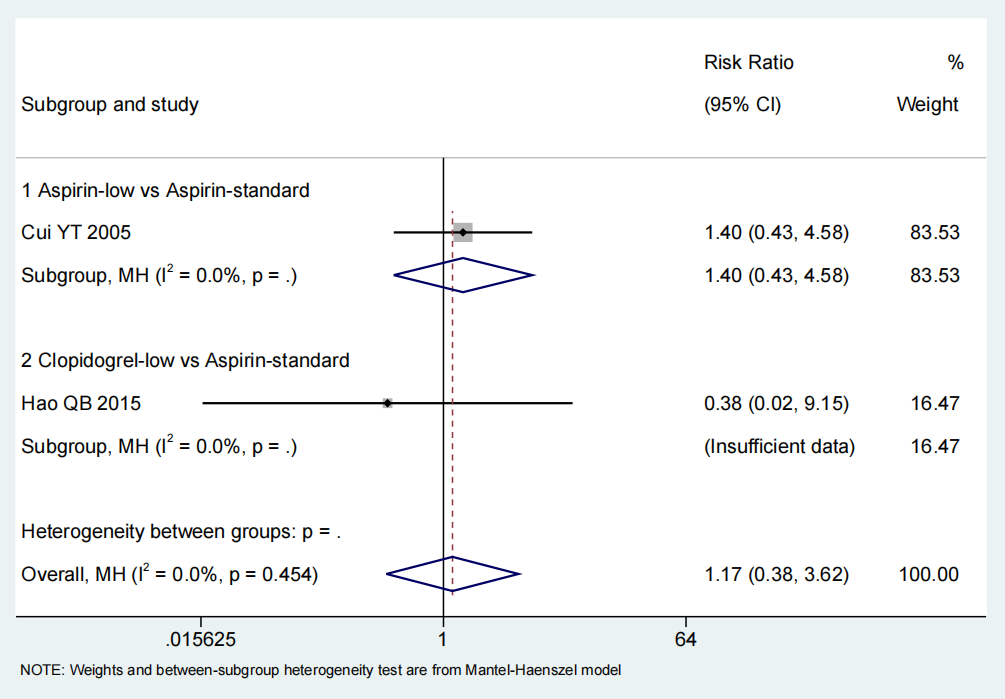


# Supplementary 6 Subgroup analysis by different countries

## 6.1 Forest for stroke by different countries


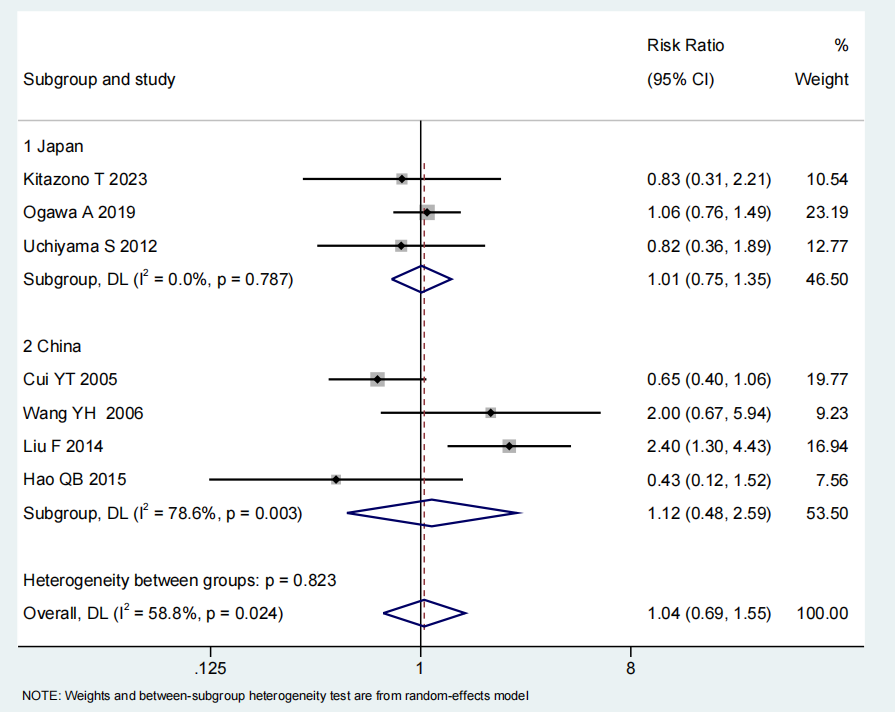


## 6.2 Forest for bleeding by different countries


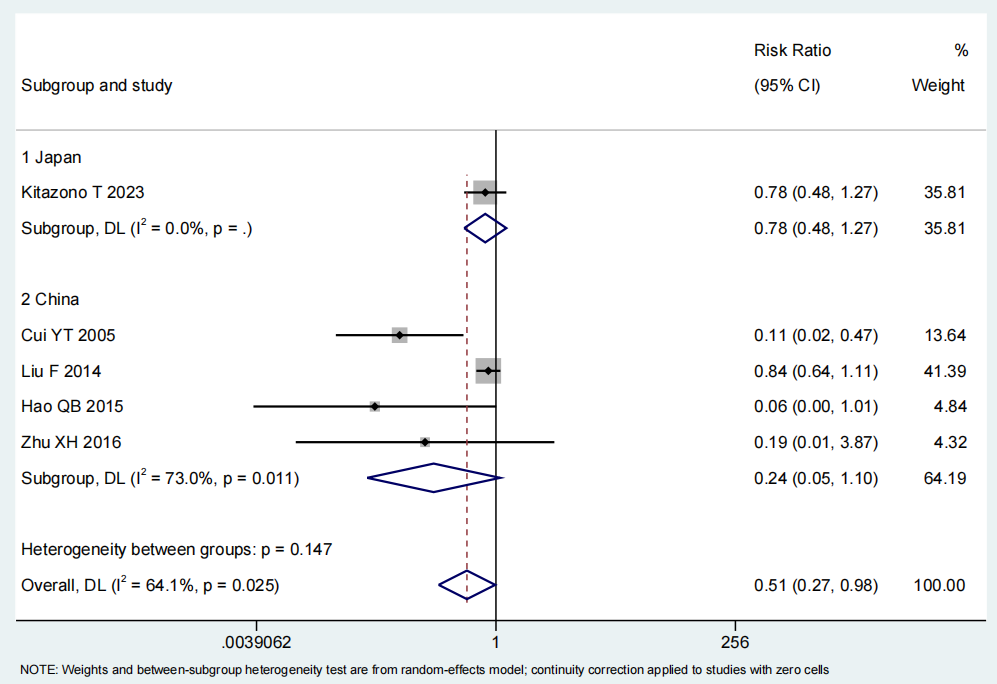


## 6.3 Forest for MI by different countries


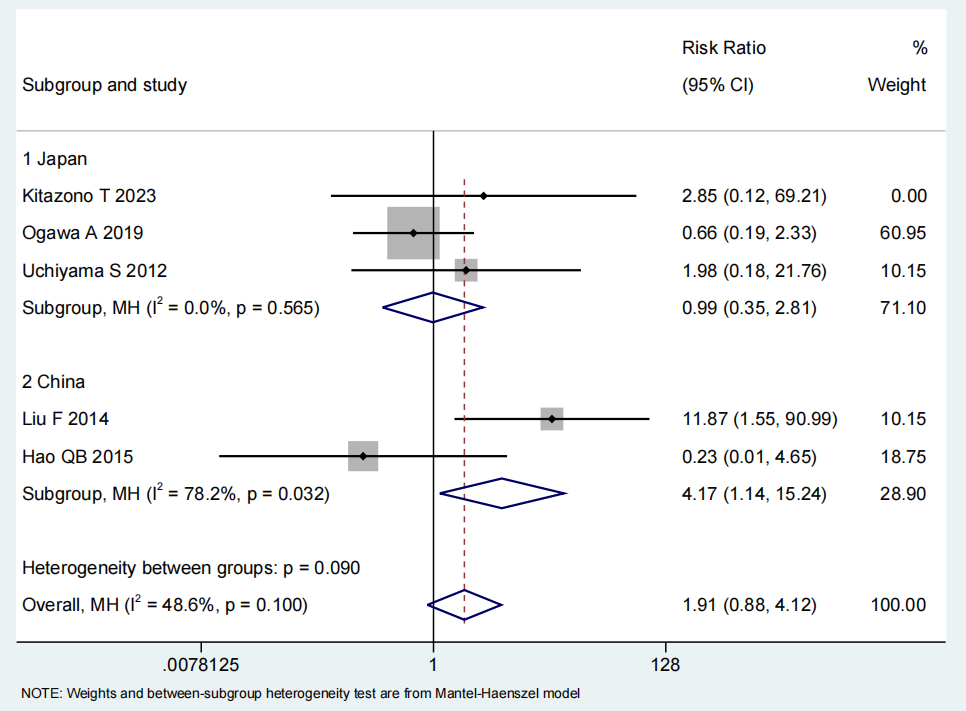


# Supplementary 7 Results of sensitive analysis

## 7.1 Sensitive analysis of stroke

## 7.2 Sensitive analysis of bleeding

## 7.3 Sensitive analysis of MI

## 7.4 Sensitive analysis of ACD

# Supplementary 8 Confunnel plots

## Confunnel plotsfor MI


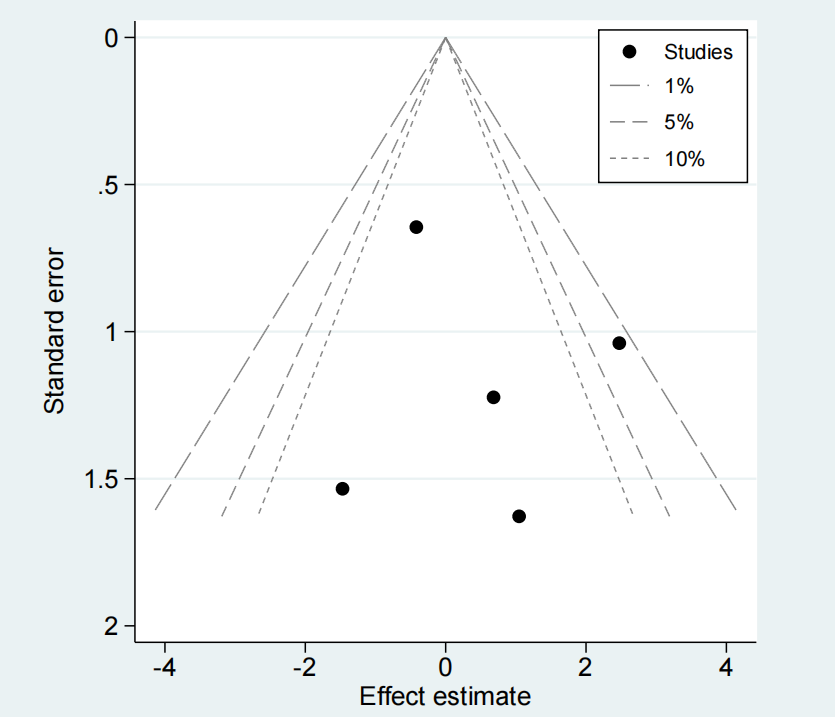

Supplement: Supplementary file 1 [file DataSheet1.docx]
